# Optimizing irrigation and nitrogen levels to achieve sustainable rice productivity and profitability

**DOI:** 10.1038/s41598-025-90464-8

**Published:** 2025-02-24

**Authors:** Mohd Salim Mir, Waseem Raja, Raihana Habib Kanth, Eajaz Ahmad Dar, Zahoor Ahmad Shah, Mohammad Anwar Bhat, Aamir Hassan Mir, Fehim Jeelani Wani, Tauseef A. Bhat, Javid Ahmad Bhat, Baby Summuna, Umer Fayaz, Suhail Fayaz, Bilal Ahmad Bhat, Nadhir Al‑Ansari, Mohamed A. Mattar, Ali Salem

**Affiliations:** 1 Division of Agronomy, Faculty of Agriculture, Sher-e-Kashmir University of Agricultural Sciences and Technology of Kashmir, Wadura, Sopore, 193201 India; 2 Krishi Vigyan Kendra, Ganderbal, Sher-e-Kashmir University of Agricultural Sciences and Technology of Kashmir, Srinagar, 190006 India; 3 West Florida Research and Education Center, University of Florida, Gainesville, FL 32565 USA; 4 Division of Agri. Extension, Faculty of Agriculture, Sher-e-Kashmir University of Agricultural Sciences and Technology of Kashmir, Wadura, Sopore, 193201 India; 5 Faculty of Horticulture, Sher-e-Kashmir University of Agricultural Sciences and Technology of Kashmir, Wadura, Sopore, 190025 India; 6 Research Centre for Residue and Quality Analysis, Faculty of Horticulture, Sher-e-Kashmir University of Agricultural Sciences and Technology of Kashmir, Wadura, Sopore, 190025 India; 7 Division of Agri. Statistics, Faculty of Agriculture, Sher-e-Kashmir University of Agricultural Sciences and Technology of Kashmir, Wadura, Sopore, 193201 India; 8 Division of Soil Science and Agricultural Chemistry, Faculty of Agriculture, Sher-e-Kashmir University of Agricultural Sciences and Technology of Kashmir, Wadura, Sopore, 193201 India; 9 Directorate of Research, Sher-e-Kashmir University of Agricultural Sciences and Technology of Kashmir, Wadura, Sopore, 193201 India; 10 Division of Genetics and Plant Breeding, Faculty of Agriculture, Sher-e-Kashmir University of Agricultural Sciences and Technology of Kashmir, Wadura, Sopore, 193201 India; 11 Department of Civil, Environmental and Natural Resources Engineering, Lulea University of Technology, 97187 Lulea, Sweden; 12 Department of Agricultural Engineering, College of Food and Agriculture Sciences, King Saud University, P.O. Box 2460, Riyadh, 11451 Saudi Arabia; 13 Structural Diagnostics and Analysis Research Group, Faculty of Engineering and Information Technology, University of Pécs, Pécs, 7622 Hungary; 14 Civil Engineering Department, Faculty of Engineering, Minia University, Minia, 61111 Egypt

**Keywords:** Rice, Irrigation regime, Nitrogen, Growth characteristics, Grain yield, Agroecology, Agroecology

## Abstract

The global scarcity of irrigation water poses a significant challenge to the sustainable production of rice and its availability worldwide. With a growing population driving increased demand for rice, it is crucial to enhance rice production while minimizing water usage. Achieving this requires a comprehensive understanding of the complex interactions between water and nitrogen dynamics and the formulation of strategies to optimize the application of irrigation water and nitrogen fertilizers. This study aims to investigate the impact of varying irrigation regimes and nitrogen application rates on rice growth attributes, yield performance, overall crop productivity, and economic returns. In the 2021 and 2022 rice growing season, two field experiments were carried out in split plot design with four nitrogen levels in sub plots [N0: Control, N1: 75% RDN (Recommended dose of nitrogen; @ 120 kg N ha^−1^), N2: 100% RDN, and N3: 125% RDN] and four irrigation treatments in main plots [I1: recommended irrigation scheduling, I2: at field capacity (20 L m^−2^), I3: 10% depletion from field capacity (20 L m^−2^), and I4: 20% depletion from field capacity (20 L m^−2^). The experiments were replicated three times. The suggested irrigation scheduling treatment (flooded) showed improved growth characteristics, such as plant height, dry matter accumulation, leaf area index, tiller count, SPAD (Soil Plant Analysis Development) value, NDVI (Normalized Difference Vegetation Index) value, leaf relative water content, and yield attributes; however, these were comparable to the application of irrigation water at field capacity. Due to improved plant growth and yield-attributing characteristics, the I1 treatment recorded the highest grain yield of 8.58 t ha^−1^ and 8.4 t ha^−1^, although it was comparable to the I2 treatment, which had grain yields of 8.27 t ha^−1^ and 8.15 t ha^−1^ in 2021 and 2022. The grain yield reported by the N3 treatment were significantly greater than those of the N2 treatment, IN 2021 and 2022 respectively. Applying nitrogen at 125% RDN (Recommended dose of nitrogen) and irrigation water at field capacity produced the highest benefit–cost ratio (1.64), which was closely followed by the same irrigation regime and 100% RDN application (1.60 BC ratio). Comparable to irrigation at field capacity, the suggested irrigation schedule demonstrated enhanced growth features, yield attributes, productivity, and profitability. The best way to achieve the optimum growth, productivity, and profitability in transplanted rice was to provide irrigation water at field capacity and nitrogen @ 100% RDN.

## Introduction

For about half of the world’s population, rice is a basic staple grain that is essential to global food security^1,2^. 517.6 million tons are produced globally, with 118 nations growing it^3,4^. With 148.3 million tonnes harvested, China is the largest producer in Asia, followed by India, which produced 120 million tonnes in 2020–2021^5^. Irrigated rice cultivation is crucial to the region’s food and livelihood security^6^. A vital part of the country’s food and livelihood security system, rice makes up 44.5 million hectares, or one-fourth of the country’s total cultivated area. It also contributes around 40–43%, or 116.42 million tonnes, of the country’s total food grain production^7^. Rice has traditionally been grown in Kashmir, which is in the North Western Himalayas, and is the main staple food consumed by the local population^8^.

Despite being intangible and susceptible to depletion, soil and water are essential natural resources for agriculture^9^. Around 85% of the freshwater resources in the world are used by agriculture, making it the biggest and most significant user of water^10^. Depending on the growth techniques used, the production of rice alone uses 3000–5000 L of water per kilogram of grain^11^. About 40% of irrigation water worldwide is used for irrigated rice, which also uses almost 80% of all irrigated freshwater resources in Asia^12^. The environmental implications of different irrigation regimes and nitrogen levels are significant and multifaceted. Inefficient irrigation practices, such as over-irrigation, can lead to water scarcity, soil salinization, and waterlogging, depleting freshwater resources, degrading soil quality and less water use efficiency. Excessive nitrogen application can result in nutrient runoff, causing water pollution, eutrophication, and contamination of groundwater with nitrates, which pose health risks and harm aquatic ecosystems. Additionally, high nitrogen levels contribute to greenhouse gas emissions, particularly nitrous oxide, exacerbating climate change. These practices can also degrade soil health, disrupt microbial communities, and reduce biodiversity, negatively affecting local ecosystems. To mitigate these impacts, sustainable practices such as efficient irrigation systems and optimum fertilization are essential for maintaining both agricultural productivity and environmental sustainability.

The future of rice production is seriously threatened by the depletion of surface and groundwater resources^13,14^. It is therefore becoming more and more difficult for rice farmers to increase water productivity by using less water to cultivate rice^15^. In light of the unavoidable effects of climate change, it is imperative that water-saving methods be adopted and promoted in rice farming in order to lower water consumption without sacrificing yields^16^. Raised bed cultivation^17^, continuous soil saturation^13^, aerobic rice systems^18^, direct-seeded rice^19^, the system of rice intensification^6^ and alternate wetting and drying^16^ are some of the water-saving techniques that have been researched and put into practice in lowland rice systems to maintain productivity while consuming less water. It has been shown that putting sustainable practices into practice, like efficient water management and soil fertility methods, can increase rice yields by up to 50% while consuming 15% less water^20^. In rice farming, the implementation of intelligent and efficient irrigation systems has demonstrated efficacy in conserving water without adversely affecting yields or other agronomic parameters^21^. These optimal management techniques can reduce water consumption by 40% and increase water production by 34% when compared to the conventional approach of continuous flooding^22^. Furthermore, water productivity has increased by 34% and irrigation water efficiency has increased by 69% as a result of intelligent irrigation water management techniques^22^. It has been demonstrated that using smart sensor-based irrigation systems for transplanted rice in India can save 13% more water than drip irrigation systems and 41% more than flooding techniques^10^. Implementing smart irrigation systems requires sensors to monitor plant, soil, and weather variables^23^. A key technique is soil moisture monitoring, often achieved by measuring soil water potential or content^24^. Monitoring soil moisture in the root zone is crucial for understanding moisture dynamics and its interaction with irrigation and plant water uptake^25^. Placing soil moisture sensors at various depths allows precise tracking of soil water content, improving irrigation efficiency and understanding crop water use^26^.

Nitrogen is vital for rice growth, development, and yield as it supports nucleotides, amino acids, and chlorophyll production^27^. Its scarcity limits rice production by affecting key physiological processes tied to biomass and grain yield. Essential for photosynthesis and crop quality^28^, nitrogen plays a critical role in global food security^29^. However, nitrogen use efficiency in rice is low due to losses from volatilization, denitrification, runoff, and leaching, especially in irrigated systems^30^. Optimizing nitrogen inputs can enhance yields, lower costs, and reduce environmental impacts^31^. There is little study on sensor-based irrigation water management for rice farming in Northern India. We postulated that sensor-based deficit irrigation could enhance crop productivity, profitability, growth characteristics, and yield qualities. An experiment was carried out to test our hypothesis. Its goals were to determine the best irrigation schedule and suitable nitrogen dosage that could be suggested for sustainable and successful rice production in the area, as well as to assess the effects of controlled irrigation and different nitrogen levels on the growth characteristics, yield attributes, productivity, and profitability of rice.

## Material and methods

### Experimental site details

At the Division of Agronomy, Faculty of Agriculture, Sher-e-Kashmir University of Agricultural Sciences and Technology of Kashmir, Wadura, Sopore, the experiment was carried out in *Kharif* 2021 and 2022 at a crop research farm. The experimental site is located at an elevation of 1590 m above mean sea level, between latitudes 34°21′ N and longitudes 74°23′ E. Before the experiment was set up, composite soil samples were taken from randomly chosen locations at soil depths of 0–10, 10–20, 20–30, 30–40, 40–60, and 60–100 cm. They were then composited and put through mechanical and chemical examination. Sand, silt, and clay contents ranged from 8.8 to 12.2%, 48.4 to 53.6%, and 34.2 to 41.3%, respectively, in the silty clay loam soil at the experimental location (Table 1). According to the field method, the saturated water content and field capacity of the various soil layers (0–10, 10–20, 20–30, 30–40, 40–60, and 60–100 cm) ranged from 0.352 to 0.484 m^3^ m^−3^ and 0.295 to 0.421 m^3^ m^−3^, respectively. Depending on the soil layer, the bulk density of the soil varied from 1.32 to 1.37 Mg m^−3^. The International Pipette Method was used to determine the texture of the soil^32^. The pycnometer and core sampler method were used to measure the bulk and particle densities of the soil^33^. The soil’s chemical examination showed that it had a neutral pH, medium levels of organic carbon, accessible nitrogen, phosphorus, and potassium, and normal electrical conductivity (Table 2).

**Table 1:** Physical characteristics of the soil of the experimental field.

Depth (cm)	Field capacity (m^3^ m^−3^)	Bulk density (Mg m^−3^)	Saturated water content (m^3^ m^−3^)	Sand (%)	Silt (%)	Clay (%)
0–10	0.295	1.37	0.352	12.2	53.6	34.2
10–20	0.337	1.36	0.386	11.9	50.8	37.3
20–30	0.361	1.35	0.418	10.1	52.3	37.6
30–40	0.397	1.36	0.445	9.2	51.7	39.1
40–60	0.414	1.33	0.472	8.8	50.5	40.7
60–100	0.421	1.32	0.484	10.3	48.4	41.3

**Table 2:** Chemical characteristics of the soil of the experimental field.

Soil property	Value	Rating	Method employed
Electrical conductivity (dSm^−1^)	0.15	Normal	1:2.5 Soil water suspension with solubridge conductivity meter^34^
pH	6.9	Neutral	1:2.5 Soil water suspension using systronics pH meter^34^
Organic carbon (%)	0.88	Medium	Wet digestion method^35^
Available N (kg ha^−1^)	380	Medium	Modified alkaline permanganate method^36^
Available P_2_O_5_ (kg ha^−1^)	18.5	Medium	Extraction with 0.5 M NaHCO_3_^37^ using systronics spectrophotometer
Available K_2_O (kg ha^−1^)	272	Medium	Extraction with neutral normal NH_4_OAC using systronics flame photometer^38^

### Climate and weather conditions

The region has a continental, Mediterranean, and temperate climate with hot summers and cold winters. Over 80% of the precipitation comes from western disturbances, and the average annual precipitation over the last 30 years is 812 mm. Both in summer and winter, there is significant variation in the minimum and maximum temperatures, which range from − 8.0 to 33 °C.

**Fig. 1:**
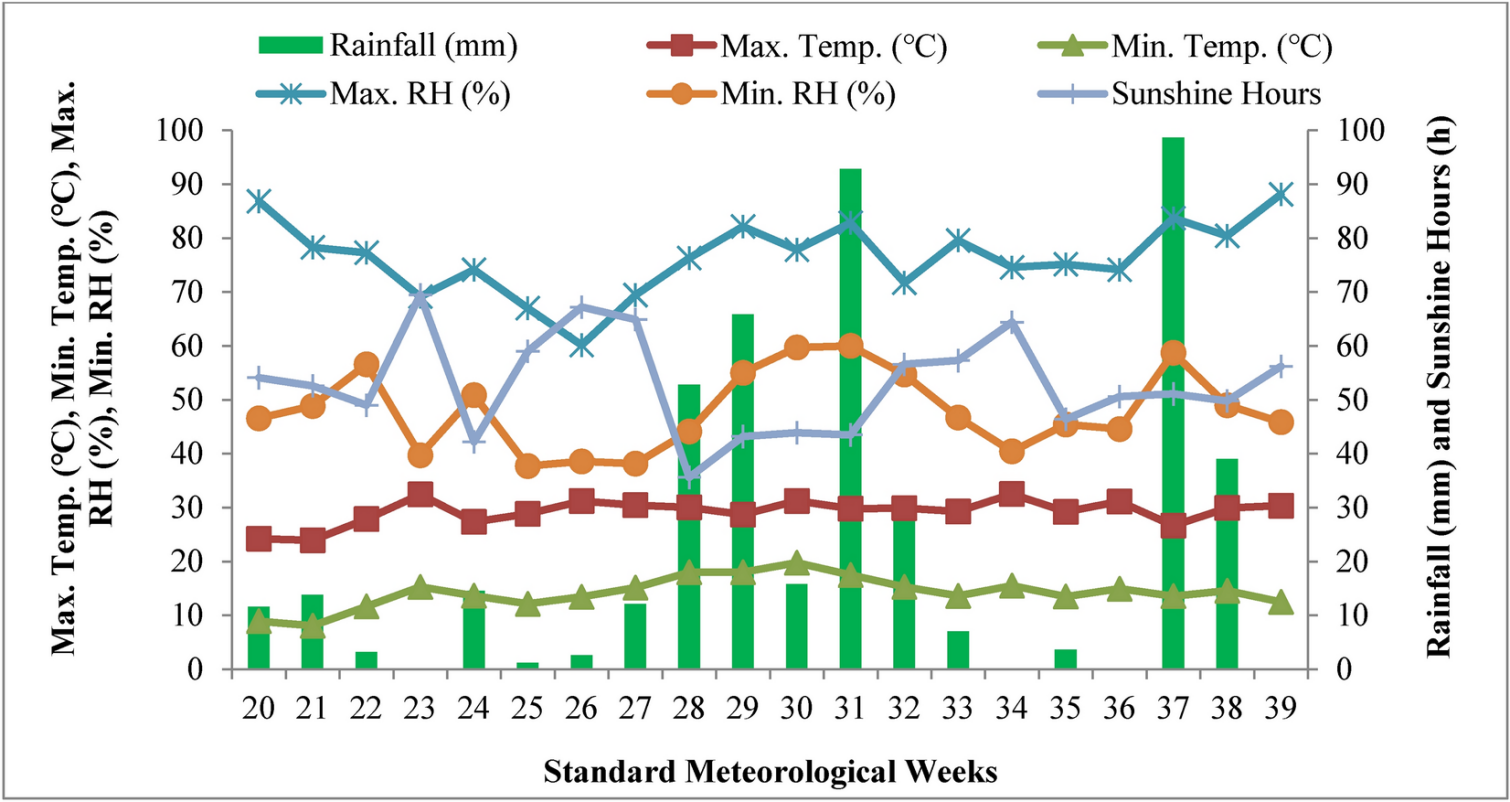
The temporal change in maximum and minimum temperature, maximum and minimum relative humidity, rainfall and sunshine hours during the 2021 growing season.

**Fig. 2:**
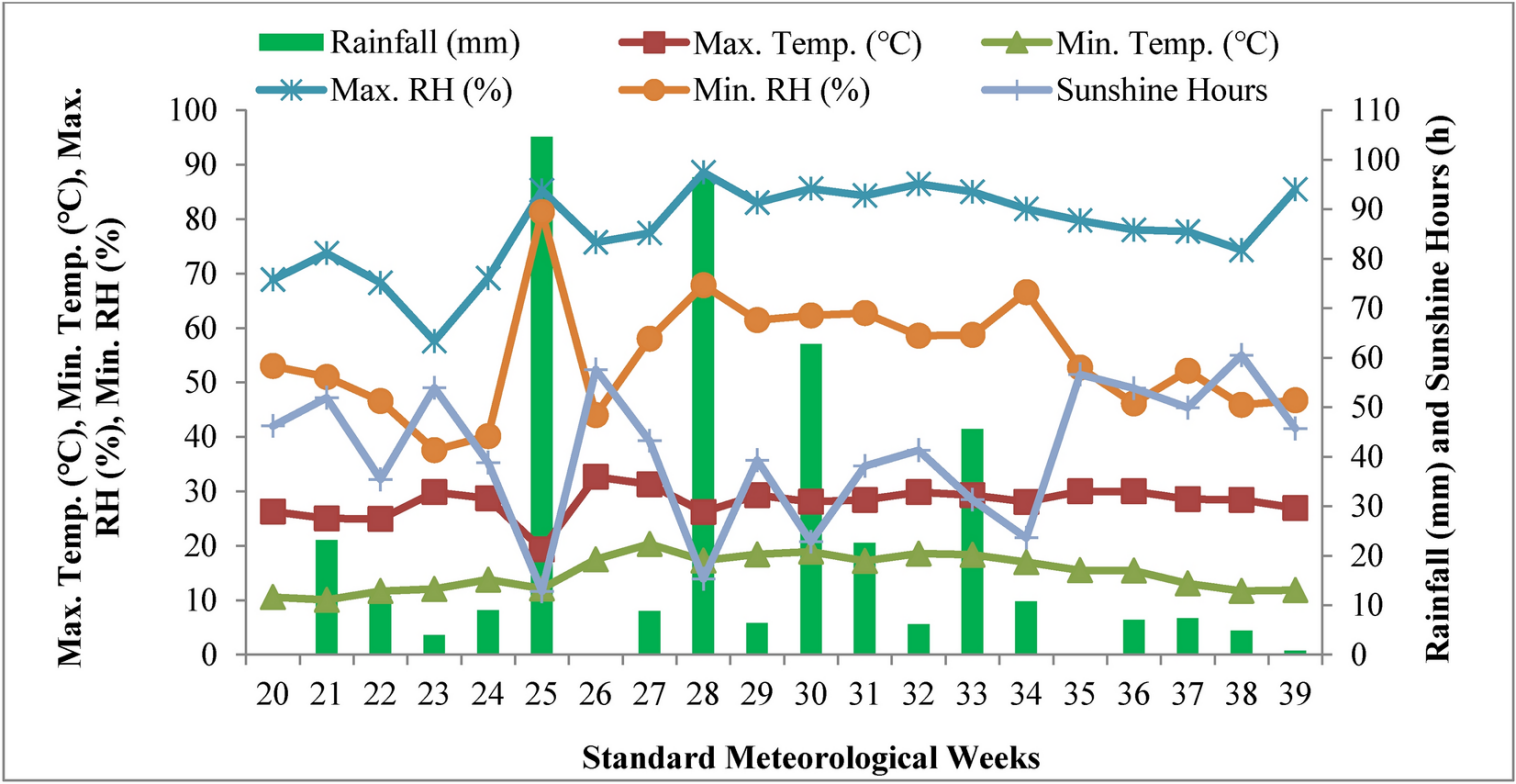
The temporal change in maximum and minimum temperature, maximum and minimum relative humidity, rainfall and sunshine hours during the 2022 growing season.

Figures 1 and 2 show the average weather data for the 2021 and 2022 cropping seasons that were collected at the Meteorological Observatory at the Division of Agronomy, Sher-e-Kashmir University of Agricultural Sciences and Technology of Kashmir, Shalimar. During the crop growing seasons of 2021 and 2022, the minimum temperature ranged between 8.1 and 19.8 °C and 10.1 and 20.3 °C, the maximum temperature ranged between 23.9 and 32.4 °C and 19.3 and 32.6 °C, and the average maximum relative humidity ranged between 60 and 88% and 57–88%. Meanwhile, the mean minimum relative humidity ranged between 38 and 60% and 37–81%, respectively. In 2021 and 2022, there were 53 and 41 sunlight hours per typical climatic week, respectively. Rainfall totals for 2021 and 2022 were 463 mm and 432 mm, respectively, throughout the testing period.

### Experimental design and treatment details

**Fig. 3:**
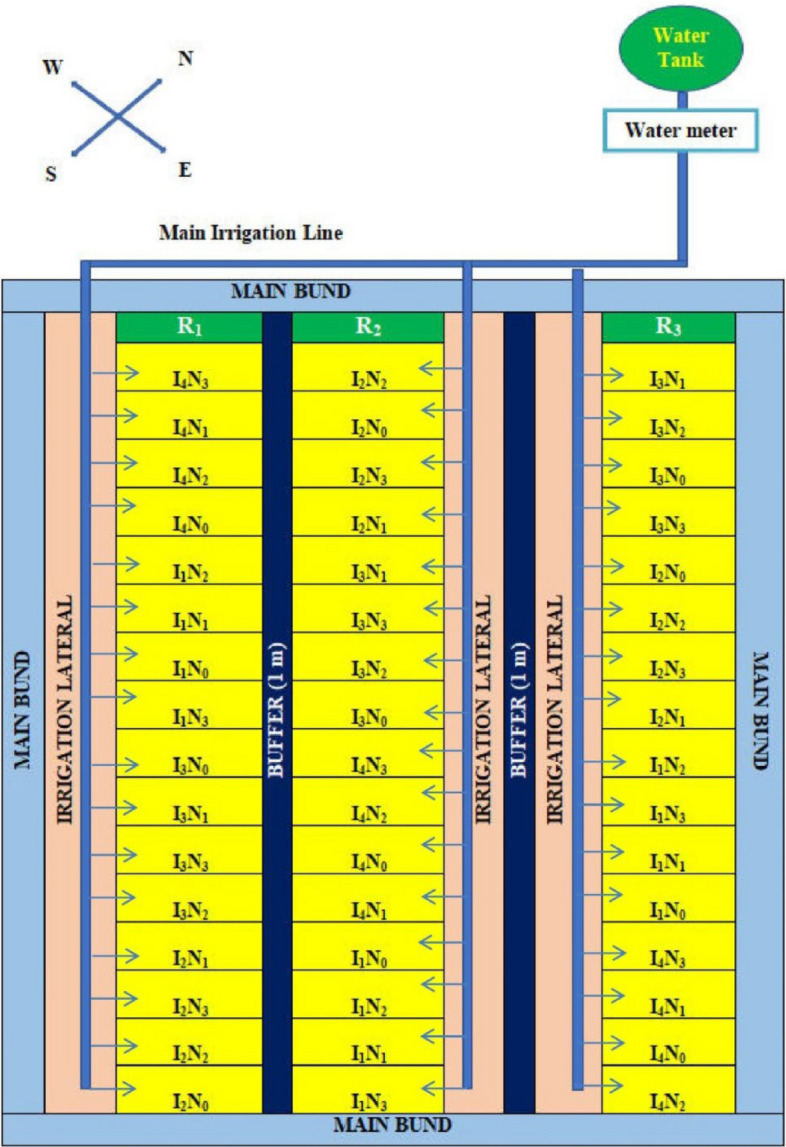
Layout plan of the experimental field.

I1: recommended irrigation scheduling, I2: at field capacity (20 L m^−2^), I3: 10% depletion from field capacity (20 L m^−2^), and I4: 20% depletion from field capacity (20 L m^−2^) were the four main plot treatments in the experiment. There were also four subplot treatments: N0: Unfertilized control, N1: 75% RDN, N2: 100% RDN (recommended dose of nitrogen; @120 kg ha^−1^), and N3: 125% RDN. To prevent water from flowing between the plots, a one-meter buffer zone was maintained between them. The blocks were also spaced one meter apart. Each experimental plot was 5 m by 3 m, and to stop water loss, bunds of 15 cm were constructed (Fig. 3).

### Crop management practices

After the previous crop was harvested, a tractor-drawn disc plough was used to plough the main field. The soil was then leveled manually using a traditional land leveler after three ploughings were made with a tiller to get it to a fine tilth. Plot paths, irrigation and drainage channels, and replication borders were all built by hand. In accordance with the treatments, the bunds were created in a dry state before the entire field was irrigated to facilitate puddling and give the bunds their final shape. Nursery beds measuring 3 m in width and 2 m in length were set up. To remove extra water, drainage canals were installed along the bed. In addition to applying the appropriate amounts of N, P, and K, a well-decomposed farm yard manure (FYM) mixture was sprayed on these nursery beds. Shalimar Rice-4 seeds were soaked for 48 h before being incubated for a further 48 h in damp gunny cloth to allow for sprouting.Shalimar Rice-4 is an early maturing and high yielding variety developed by Mountain Research Centre for Field Crops (MRCFC) Khudwani of SKUAST-K and was released in 2017. It is a cold tolerant indica variety having resistance to blast, erect plant type, light green basal leaf sheath, high test weight, high biomass yield and easy threshability. It is highly recommended for cultivation in irrigated low lands of the Kashmir valley (up to 1700 m amsl). Its yield potential is 8.5–9.0 t ha^−1^ and matures in 135–140 days. The seeds were dispersed evenly across the nursery beds at a seed rate of 60 kg ha^−1^. Channels were created around the nursery beds, and water was regularly let into them. Tricyclazole 75 WP @ 0.6 g kg^−1^ seed was applied to the seeds to stop seed rot, pre-emergence damping off, and rice blast. Following appropriate puddling of plots, with 4–5 seedlings per hill at a geometry of 15 cm × 15 cm between the hills, the transplantation of 30-day-old seedlings was carried out on June 17, 2021, and June 19, 2022. When the field was laid out, experimental locations received 10 t ha^−1^ of FYM. Before transplanting, the entire required dose of potassium and phosphorus was evenly administered to each plot as a basal dose using di-ammonium phosphate and muriate of potash at rates of 60 and 30 kg P_2_O_5_ and K_2_O ha^−1^, respectively. Using urea, nitrogen was administered in accordance with treatments, with half going to the basal and the other half going to the tillering (18–22 DAT) and panicle initiation stages (35–40 DAT) phases. Four days following transplantation, a hand broadcast of bensulfuron methyl + pretilachlor (60 g + 600 g a.i. ha^−1^) under the trade name Erase @ 10 kg ha^−1^ was applied to control weeds. Manual hand weeding was done 15 days after herbicide application.

### Irrigation management

Two 7000-L PVC tanks were installed at a height of two meters, and a 1.5-inch water meter was installed at the tank’s outflow. A surface watering system was put in place prior to transplanting, and it involved a PVC conduit that ran parallel to the plots. In order to ensure that one plot at a time within each replication was irrigated, each plot had a centrally positioned inlet with a watertight butterfly valve. A 1.5 hp pump was built at the water tank’s outlet behind the water meter to maintain the pipeline’s pressure. To supply water to the two PVC tanks, a second 2.0 hp pump was placed 20 feet from the water source. For every treatment, irrigation water was used until the required amount arrived.

The measurement of volumetric soil moisture was conducted through the use of the Delta-T Devices PR2 soil moisture profile probe (Delta-T Device, UK), with generalised calibrations done as per the PR2 profile probe user manual (https://delta-t.co.uk/wp-content/uploads/2017/02/PR2_user_manual_version_5.0.pdf) Access tubes were installed to a depth of 1 m in the soil for this purpose. Soil moisture levels were assessed at six different depths (0–10, 10–20, 20–30, 30–40, 40–60, and 60–100 cm) both prior to and following each irrigation application in all treatments. The amount of irrigation water administered to these treatments was recorded using a water meter installed in the main line. The amount of irrigation applied to each treatment is given in supplementary Table 1.

### Biometric crop observations

Plant height was measured from the surface to the tip of the extreme fully opened leaf during the vegetative growth phase and up to the apex of the panicle upon flowering on 10 randomly chosen plants from each treatment at periodic intervals of the crop. The heights were averaged and expressed in centimetres. For the estimation of dry matter of plant samples, a quadrant of 0.25 m^2^ was used. Plant samples were taken at periodic intervals and dried under sunlight for 3–4 days before being dried in an oven for 48 h at 60–65 °C to achieve a stable weight. Tiller count (through use of quadrant) was taken at 15 days interval till harvest from every experimental plot and were averaged and expressed in no. m^−2^. SPAD value was recorded at 15 days interval upto 75 DAT using SPAD meter. The SPAD-502Plus (Soil and plant analysis development) chlorophyll meter (Konica Minolta) was used for recording rice leaf chlorophyll units. Normalized Difference Vegetation Index (NDVI) value was recorded at 15 days interval upto 75 DAT using Green Seeker handheld crop sensor (Trimble Agriculture, USA) for crop health.

Leaf area meter was used for measuring leaf area (cm^2^/plant) (Systronics, 211) from same plants. For this, whole leaves were taken out from the stem and cleaned thoroughly with normal tap water and then de-ionized water followed by drying with the help of tissue paper. Leaf area index is defined as the ratio of leaf surface to the ground area occupied by the plant and was calculated by the formula as per^39^.

1 $$\text{Leaf area index }=\frac{\text{Total leaf area}}{\text{Ground area}}$$

Leaf relative water content was recorded at panicle initiation, flowering and milking stages and was computed by using the formula: measure of plant water status which reflects the metabolic activity of plant tissues and used as a significant index for dehydration tolerance. RWC was measured on the basis of oven dry weight method given by Weatherly (1950) at panicle initiation, 50% anthesis and milking stage. Leaf samples were taken at 2 pm when there was maximum temperature in the day. After collection, leaf sample was kept in to air tight polybags to prevent the transpiration. In laboratory, leaves were weighed for their fresh weight and cut into 1–2 cm pieces. This fresh leaf material was kept in double distilled water in a petridish for 2 h to make the leaf tissue turgid. The leaf pieces were weighed for their turgid weight. After taking the turgid weight leaf materials were kept in a butter paper bag and dried in oven at 65 °C for 24 h till constant weight was achieved and their dry weights were recorded. The RWC was calculated by using the formula

2 $$\text{RWC }\left(\text{\%}\right)=\frac{(\text{W}-\text{DW})}{(\text{TW}-\text{DW})}\times 100$$

where W = Sample fresh weight (g), DW = Sample dry weight (g) and TW = Sample turgid weight (g).

Ten randomly selected panicles from each treatment were assessed for panicle length, panicle weight, and filled grains per panicle, which were then averaged and represented in centimetres, grams, and grains per panicle, respectively. Grain samples were taken from each plot and dried properly at the time of threshing and from each of the collected samples; 1000 grains were taken for test weight and expressed in grams. From each net plot grain yield, straw yield, and biological yield was computed and by using the following formula harvest index was calculated

3 $$\text{Harvest index }=\frac{\text{Economic yield}}{\text{Biological yield}} \times 100$$

The relative economics was calculated based on grain and straw yield per hectare, to test the relative benefits of different treatments. As per the ongoing market rates, both input as well as output cost was estimated. The benefit cost (B:C) ratio (returns on unit rupee invested) was expressed as:

4 $$Benefit:CostRatio =\frac{NetReturns}{Total\,cost\,of\,cultivation}$$

### Statistical analysis

The OPSTAT software (https://opstat.somee.com/opstat/) was used to perform the analysis of variance and test significance of irrigation and nitrogen. The variables tested were plant height, LAI, tiller count, SPAD, NDVI, leaf relative water content, panicle density, panicle length, panicle weight, spikelets per panicle, filled grains per panicle, test weight, grain yield, biological yield and harvest index. A critical difference test at α = 0.05 was used to separate the means. Regression analysis in Microsoft Excel (https://office.microsoft.com/excel) was used to investigate the functional relationship between applied irrigation amount and growth and yield variables in each year. The best fit was selected based on R^2^ values. Graphs were designed using Microsoft Excel.

## Results

### Plant height

Plant height of rice was significantly (*p* ≤ 0.05) influenced by irrigation schedules and nitrogen levels during both the years of experimentation. It was found that plant height increased exponentially up to 75 DAT and then increased at a decreasing rate until maturity. Among the variable irrigation schedules, recommended irrigation scheduling (RIS) recorded significantly higher periodic plant height but was at par with the application of irrigation water at field capacity (I2) as compared to 10% depletion from field capacity (I3) and 20% depletion from field capacity (I4) treatments during both the years (2021 and 2022) of experimentation. The highest plant height of 123.6 cm and 121.1 cm was found in RIS followed by field capacity treatment which recorded the plant height of 121.5 cm and 119.2 cm at harvest during 2021 and 2022 respectively whereas the lowest plant height of 110.4 cm and 107.4 cm at harvest was recorded with the application of irrigation water at 20% depletion from field capacity respectively during both the years of experimentation. The percentage increase in plant height in RIS was 6.47% and 6.85% and application of irrigation water at field capacity was 4.85% and 5.36% as compared to 10% depletion from field capacity, during 2021 and 2022 respectively. Among the different nitrogen levels, 125% RDN (recommended dose of nitrogen) recorded the highest plant height; however, it was at par with 100% RDN treatment throughout the crop growth period during both the years of experimentation. 125% RDN recorded significantly higher plant height of 124.3 cm and 121.7 cm at maturity followed by 100% RDN treatment which recorded the plant height of 121.7 cm and 118.1 cm during 2021 and 2022 respectively. The percentage increase in plant height at maturity with the application of nitrogen @ 125% RDN was 6.35% and 5.91% and @ 100% RDN was 4.35% and 3.04% as compared to 75% RDN during 2021 and 2022 respectively. Moreover, it was found that (N0) control treatment recorded significantly lowest periodic plant height during both the years of experimentation. The interaction effect between irrigation schedules and nitrogen levels were found non-significant.

### Dry matter accumulation

**Fig. 4:**
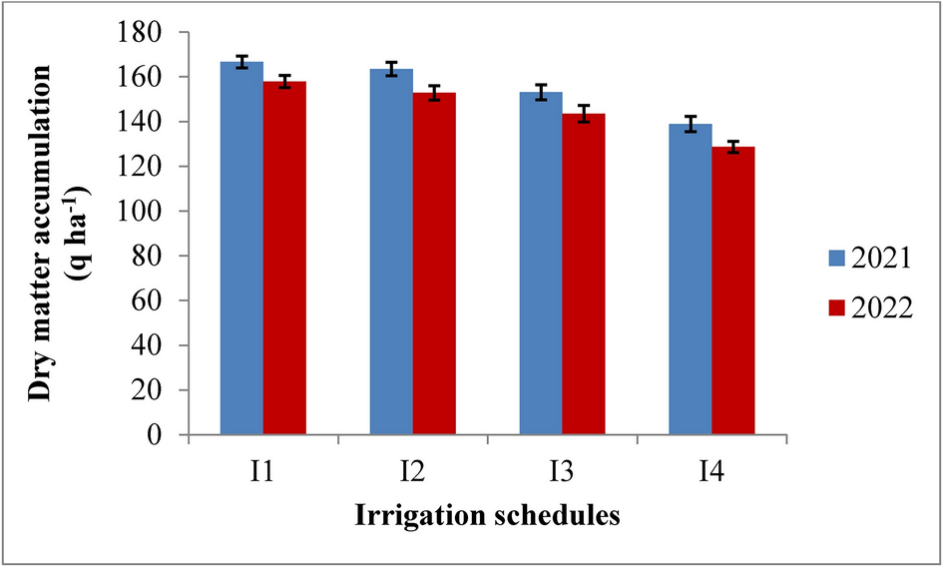
Dry matter accumulation of rice as influenced by variable irrigation schedules (I1: recommended irrigation scheduling; I2: application of irrigation water at field capacity; I3: application of irrigation water at 10% depletion from field capacity; I4: application of irrigation water at 20% depletion from field capacity). The vertical bars represent the mean ± SEm values.

**Fig. 5:**
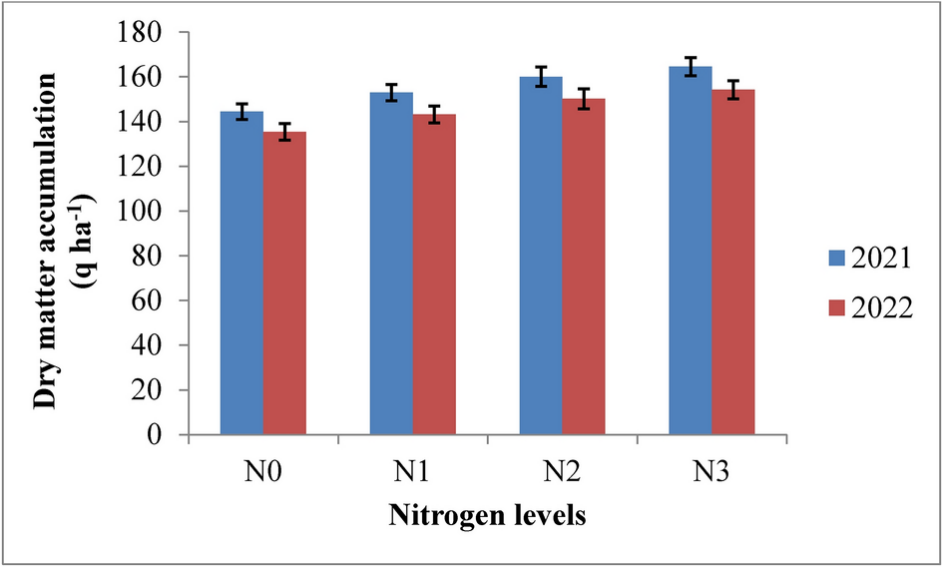
Dry matter accumulation of rice as influenced by varying nitrogen levels. {(N0: Control; N1: 75% RDN (Recommended dose of nitrogen); N2: 100% RDN; N3: 125% RDN}. The vertical bars represent the mean ± SEm values.

Dry matter accumulation in rice was significantly affected by irrigation schedules and nitrogen levels. It increased rapidly up to 75 days after transplanting (DAT) and then slowed until maturity in both years (Table 3 and Figs. 4 and 5). Among the various irrigation treatments, RIS showed the highest dry matter accumulation, although it was statistically similar to the irrigation applied at field capacity (I2). Both were superior to the 10% depletion from field capacity (I3) and 20% depletion from field capacity (I4) treatments during 2021 and 2022. The highest dry matter accumulation, recorded under the recommended irrigation schedule, was 166.58 q ha^−1^ in 2021 and 157.87 q ha^−1^ in 2022. This was followed by the field capacity treatment, which showed dry matter values of 163.41 q ha^−1^ and 152.82 q ha^−1^ at harvest in the respective years. The lowest accumulation, at 138.85 q ha^−1^ and 128.66 q ha^−1^, was observed in the 20% depletion treatment (I4). The recommended irrigation schedule resulted in an 8.14% and 9.13% increase in dry matter accumulation in 2021 and 2022, respectively, while the field capacity treatment showed increases of 6.35% and 6.13% compared to the 10% depletion treatment in the same years. Regarding nitrogen levels, the application of 125% recommended dose of nitrogen (RDN) led to the highest dry matter accumulation, though it was statistically similar to the 100% RDN treatment throughout the crop growth period in both years. The 125% RDN treatment produced dry matter accumulations of 164.50 q ha^−1^ and 154.20 q ha^−1^ at maturity, followed by the 100% RDN treatment, which resulted in 160.03 q ha^−1^ and 150.10 q ha^−1^ in 2021 and 2022, respectively. Dry matter accumulation increased by 7.02% and 7.15% with 125% RDN and by 4.43% and 4.62% with 100% RDN compared to 75% RDN in both years. The control (N0) showed the lowest accumulation, with no significant interaction between irrigation and nitrogen levels.

**Table 3:** Influence of variable irrigation schedules and nitrogen levels on dry matter accumulation (q ha^−1^) of rice.

Treatments	15 DAT		30 DAT		45 DAT		60 DAT		75 DAT		90 DAT		105 DAT		Maturity	
	2021	2022	2021	2022	2021	2022	2021	2022	2021	2022	2021	2022	2021	2022	2021	2022
Irrigation schedules																
I_1_	1.38	1.34	10.53	10.44	57.18	55.02	121.20	118.79	145.72	140.33	157.55	151.12	163.61	156.55	166.58	157.87
I_2_	1.33	1.28	10.52	10.27	55.53	53.84	118.77	114.18	142.18	135.31	154.44	146.19	160.50	151.50	163.41	152.82
I_3_	1.10	1.12	9.89	9.78	47.96	45.11	107.70	104.46	131.95	125.92	144.01	136.73	150.06	142.07	153.02	143.45
I_4_	0.98	0.96	8.40	8.30	44.55	42.80	93.47	89.91	117.78	111.23	129.87	121.97	135.86	127.41	138.85	128.66
SE (m) ±	0.02	0.03	0.16	0.12	0.47	0.42	1.40	1.37	1.42	1.43	1.47	1.45	3.11	3.70	2.22	2.85
C.D. (*p* ≤ 0.05)	0.07	0.09	0.42	0.40	1.68	1.43	4.93	4.83	5.01	5.06	5.20	5.13	10.99	13.05	7.84	10.06
Nitrogen levels																
N_0_	1.11	1.07	9.41	9.18	46.32	44.30	102.82	99.54	126.99	121.01	139.13	131.68	141.40	134.06	144.40	135.35
N_1_	1.17	1.17	9.59	9.50	49.47	47.52	106.68	103.23	130.56	124.61	142.51	135.48	149.91	141.81	152.94	143.16
N_2_	1.23	1.21	10.00	9.88	53.62	51.56	113.79	110.30	137.91	131.71	149.89	142.50	157.12	148.85	160.03	150.10
N_3_	1.29	1.25	10.33	10.23	55.82	53.40	117.85	114.26	142.16	135.44	154.33	146.35	161.61	152.80	164.50	154.20
SE(m) ±	0.01	0.02	0.14	0.12	0.97	0.92	1.39	1.28	1.39	1.23	1.53	1.34	2.85	2.28	2.62	2.54
C.D. (*p* ≤ 0.05)	0.05	0.07	0.39	0.35	2.86	2.72	4.12	3.98	4.34	3.81	4.49	3.88	8.38	6.70	7.71	7.46

{I1: recommended irrigation scheduling; I2: application of irrigation water at field capacity; I3: application of irrigation water at 10% depletion from field capacity; I4: application of irrigation water at 20% depletion from field capacity; N0: Control; N1: 75% RDN (Recommended dose of nitrogen); N2: 100% RDN; N3: 125% RDN}.

### Leaf area index

The analysis of the data revealed that the leaf area index (LAI) of rice was significantly influenced by both irrigation schedules and nitrogen levels during the two years of the study. It was observed that the LAI increased rapidly up to 60 days after transplanting (DAT) before gradually declining as the crop approached maturity in both years. Among the various irrigation treatments, the RIS treatment resulted in the highest LAI, although it was statistically on par with irrigation applied at field capacity (I2), compared to the 10% depletion (I3) and 20% depletion (I4) from field capacity treatments in both 2021 and 2022. The highest LAI values of 4.90 and 4.85 were recorded under the recommended irrigation schedule at 60 DAT, followed by the field capacity treatment, which showed values of 4.67 and 4.62 during the same period in 2021 and 2022, respectively. The lowest LAI values, 3.66 and 3.62 at 60 DAT, were observed under the 20% depletion treatment in both years. The data revealed that nitrogen levels significantly influenced the Leaf Area Index (LAI) during both years of the study. The application of 125% of the recommended nitrogen dose (RDN) produced the highest LAI, though it remained statistically comparable to the 100% RDN treatment across the crop growth period in both years. The 125% RDN treatment achieved LAI values of 4.60 and 4.48 at 60 DAT, followed closely by the 100% RDN treatment, which recorded 4.46 and 4.33 in 2021 and 2022, respectively. The control treatment (N0) consistently showed the lowest LAI across both years.

### Tiller count

**Fig. 6:**
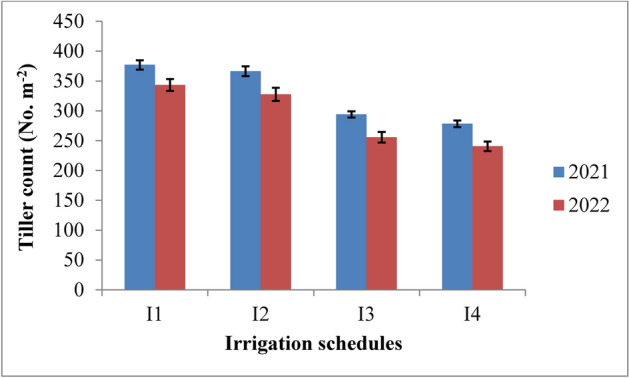
Tiller count of rice as influenced by variable irrigation schedules (I1: recommended irrigation scheduling; I2: application of irrigation water at field capacity; I3: application of irrigation water at 10% depletion from field capacity; I4: application of irrigation water at 20% depletion from field capacity). The vertical bars represent the mean ± SEm values.

**Fig. 7:**
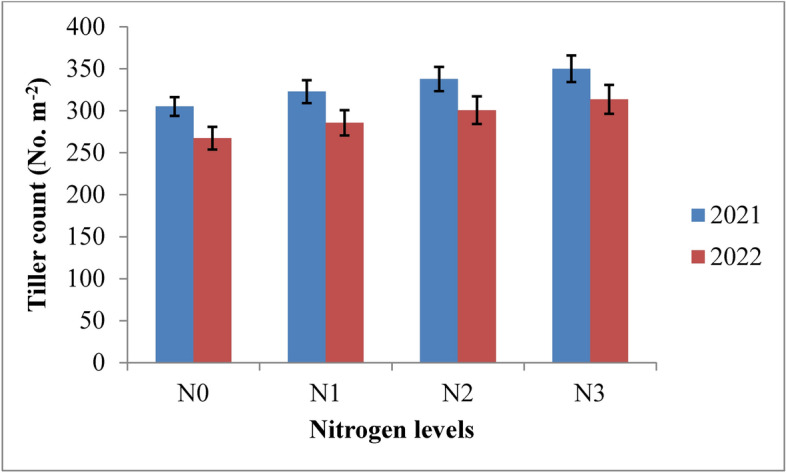
Tiller count of rice as influenced by varying nitrogen levels. {(N0: Control; N1: 75% RDN (Recommended dose of nitrogen); N2: 100% RDN; N3: 125% RDN}. The vertical bars represent the mean ± SEm values.

The tiller count in rice was significantly influenced by irrigation schedules and nitrogen levels during both years of the study. Tiller count increased up to 60 days after transplanting (DAT) and then decreased until maturity (Table 4 and Figs. 6 and 7). Among the irrigation treatments, the recommended irrigation schedule (I1) resulted in the highest tiller count, though it was statistically similar to irrigation at field capacity (I2). Both were superior to the 10% depletion (I3) and 20% depletion (I4) treatments during 2021 and 2022. The highest tiller count was recorded under the RIS, with 459.7 and 445.1 tillers at 60 DAT in 2021 and 2022, respectively, followed by the field capacity treatment (455.0 and 422.1 tillers). The lowest tiller count was observed under the 20% depletion treatment, with 387.3 and 371.7 tillers at 60 DAT in 2021 and 2022, respectively. At harvest, the recommended irrigation schedule increased tiller count by 22.04% and 22.69%, while the field capacity treatment showed a 19.76% and 20.36% increase compared to the 10% depletion treatment over two years. The highest tiller count was achieved with 125% RDN, though it was statistically similar to 100% RDN in both years. The 125% RDN treatment recorded 448.5 and 425.7 tillers at 60 DAT, followed by the 100% RDN treatment with 436.4 and 414.5 tillers in 2021 and 2022, respectively. The increase in tiller count at harvest with 125% RDN was 7.8% and 8.19%, while with 100% RDN, it was 4.44% and 4.59% compared to the 75% RDN treatment in both years. The control treatment (N0) consistently recorded the lowest tiller count.

**Table 4:** Influence of variable irrigation schedules and nitrogen levels on tiller count (No. m^−2^) of rice.

Treatments	15 DAT		30 DAT		45 DAT		60 DAT		75 DAT		90 DAT		105 DAT		Maturity	
	2021	2022	2021	2022	2021	2022	2021	2022	2021	2022	2021	2022	2021	2022	2021	2022
Irrigation schedules																
I_1_	302.6	296.9	367.1	357.8	418.9	409.0	459.7	445.1	440.9	423.8	422.0	406.6	402.6	391.6	377.0	368.3
I_2_	288.9	277.4	359.1	347.6	412.4	398.6	455.0	422.1	426.3	399.4	405.3	387.0	382.0	372.3	366.3	357.5
I_3_	273.1	263.5	332.3	322.7	377.0	363.7	397.8	382.3	374.4	351.1	347.4	332.4	323.6	310.1	293.9	284.7
I_4_	262.9	257.3	311.6	302.6	365.8	350.4	387.3	371.7	362.5	334.6	331.8	316.3	307.7	293.7	278.1	269.4
SE(m) ±	4.23	5.88	3.59	3.48	5.57	3.49	4.11	7.33	6.68	8.38	6.54	4.13	6.84	6.37	3.49	5.14
C.D. (*p* ≤ 0.05)	14.93	20.76	12.68	12.27	19.65	12.31	14.51	25.88	19.53	24.99	18.13	14.59	24.13	22.48	12.33	18.15
Nitrogen levels																
N_0_	250.5	246.2	312.3	311.2	364.3	360.3	393.0	381.2	371.0	363.8	345.4	342.03	324.2	317.9	304.9	295.2
N_1_	281.2	270.7	343.7	330.6	388.8	372.6	421.9	399.9	397.1	382.4	369.2	356.13	349.9	335.8	322.7	313.5
N_2_	293.3	284.7	352.7	340.1	404.1	387.9	436.4	414.5	412.1	397.3	384.1	371.45	364.9	351.1	337.7	328.6
N_3_	302.5	293.5	361.4	348.7	417.0	400.8	448.5	425.7	423.9	409.4	395.9	382.90	376.9	362.9	350.0	341.5
SE(m) ±	6.96	6.39	5.53	5.76	4.07	3.27	4.75	5.15	4.31	4.96	5.19	4.20	8.00	5.07	5.17	4.99
C.D. (*p* ≤ 0.05)	20.45	18.78	16.25	16.91	12.95	9.68	13.95	15.12	12.67	14.58	15.12	12.35	23.49	14.90	15.20	14.67

{I1: recommended irrigation scheduling; I2: application of irrigation water at field capacity; I3: application of irrigation water at 10% depletion from field capacity; I4: application of irrigation water at 20% depletion from field capacity; N0: Control; N1: 75% RDN (Recommended dose of nitrogen); N2: 100% RDN; N3: 125% RDN}.

### SPAD value

Rice SPAD values were significantly affected by both irrigation schedules and nitrogen levels throughout the study. The values increased as the crop grew, reaching their maximum at 60 days after transplanting (DAT) before decreasing in both years. Among the irrigation treatments, the RIS (I1) produced the highest SPAD values, although it was statistically similar to irrigation at field capacity (I2). Both treatments outperformed the 10% (I3) and 20% (I4) depletion from field capacity treatments in 2021 and 2022. The RIS recorded the highest SPAD values of 49.08 and 48.59 at 60 DAT in 2021 and 2022, respectively, followed by the field capacity treatment, which had SPAD values of 49.03 and 48.56. The lowest SPAD values of 46.17 and 44.81 were observed under the 20% depletion treatment at 60 DAT in both years. The data further showed that nitrogen levels had a significant effect on SPAD values. The application of 125% of the recommended dose of nitrogen (RDN) resulted in the highest SPAD values, though it was statistically similar to the 100% RDN treatment across the crop’s growth stages during both years. The 125% RDN treatment produced SPAD values of 48.54 and 47.69 at 60 DAT, followed by the 100% RDN treatment, which recorded SPAD values of 48.12 and 47.27 in 2021 and 2022, respectively. The lowest SPAD values were consistently recorded in the control treatment (N0) across both years.

### NDVI (Normalized difference vegetation index) value

The NDVI value of rice was significantly influenced by irrigation schedules and nitrogen levels during both years of the study. NDVI values increased with crop growth, peaking at 60 days after transplanting (DAT) and then declined as the crop matured. The recommended irrigation schedule (I1) yielded the highest NDVI values, comparable to the field capacity treatment (I2), and outperformed the 10% (I3) and 20% (I4) depletion treatments in both 2021 and 2022. At 60 DAT, the recommended irrigation recorded peak NDVI values of 0.758 and 0.735 in 2021 and 2022, respectively, followed by the field capacity treatment with values of 0.753 and 0.730. The lowest NDVI values, 0.679 and 0.617 at 60 DAT, were observed under the 20% depletion treatment in both years. Nitrogen levels also had a significant impact on NDVI values. The application of 125% of the recommended dose of nitrogen (RDN) resulted in the highest NDVI values, though it was statistically comparable to the 100% RDN treatment throughout the crop growth period in both years. The 125% RDN treatment recorded NDVI values of 0.745 and 0.708 at 60 DAT, followed by the 100% RDN treatment, which recorded values of 0.737 and 0.697 in 2021 and 2022, respectively. The control treatment (N0) consistently exhibited the lowest NDVI values across both years.

### Leaf relative water content

RWC of rice was significantly affected by irrigation schedules and nitrogen levels during both the years of experimentation. It was found that RWC of rice was highest at panicle initiation stage and thereafter declined at 50% flowering and milking during both the years of experimentation (Table 5). Among the variable irrigation schedules, RIS (I1) recorded significantly highest RWC but was at par with the application of irrigation water at field capacity (I2) as compared to 10% depletion from field capacity (I3) and 20% depletion from field capacity (I4) treatments during both the years (2021 and 2022) of experimentation. The highest RWC (%) of 86.79 and 85.46 was found in recommended irrigation scheduling followed by field capacity treatment which recorded the RWC of 85.30 and 84.09 at panicle initiation stage during 2021 and 2022 respectively whereas the lowest RWC of 78.38 and 75.12 at panicle initiation stage was recorded with the application of irrigation water at 20% depletion from field capacity respectively during both the years of experimentation. The percentage increase in RWC at panicle initiation stage in RIS was 6.55% and 7.46% and application of irrigation water at field capacity was 4.92% and 5.95% as compared to 10% depletion from field capacity treatment during 2021 and 2022 respectively. The highest Relative Water Content (RWC) was observed with 125% RDN, comparable to 100% RDN at panicle initiation, 50% flowering, and milking stages during both years. At panicle initiation, 125% RDN recorded RWCs of 84.38 and 82.55, while 100% RDN showed 83.43 and 81.60 in 2021 and 2022, respectively. The RWC increase at 125% RDN was 2.18% and 2.20%, and at 100% RDN was 1.04% and 1.06%, compared to 75% RDN. The control (N0) had the lowest RWC, and no significant interaction was found between irrigation and nitrogen levels.

**Table 5:** Influence of variable irrigation schedules and nitrogen levels on leaf relative water content (RWC %) of rice.

Treatments	Panicle initiation		50% Flowering		Milking	
	2021	2022	2021	2022	2021	2022
Irrigation schedules						
I_1_	86.79	85.46	82.70	85.40	75.42	74.07
I_2_	85.30	84.09	80.71	84.64	73.79	72.55
I_3_	81.10	79.08	76.41	80.14	70.35	68.38
I_4_	78.38	75.12	73.37	78.57	68.73	66.52
SE(m) ±	0.46	0.58	0.65	0.75	0.87	0.61
C.D. (*p* ≤ 0.05)	1.63	1.81	2.30	2.65	3.09	2.18
Nitrogen levels						
N_0_	81.19	78.86	75.19	79.56	69.74	67.97
N_1_	82.56	80.73	78.13	81.42	71.92	70.24
N_2_	83.43	81.60	79.16	82.80	72.70	71.03
N_3_	84.38	82.55	80.72	84.97	73.93	72.27
SE(m) ±	0.46	0.53	0.91	0.77	0.62	0.67
C.D. (*p* ≤ 0.05)	1.36	1.52	2.69	2.18	1.82	1.92

{I1: recommended irrigation scheduling; I2: application of irrigation water at field capacity; I3: application of irrigation water at 10% depletion from field capacity; I4: application of irrigation water at 20% depletion from field capacity; N0: Control; N1: 75% RDN (Recommended dose of nitrogen); N2: 100% RDN; N3: 125% RDN}.

### Yield attributing characters

#### Panicle density

**Fig. 8:**
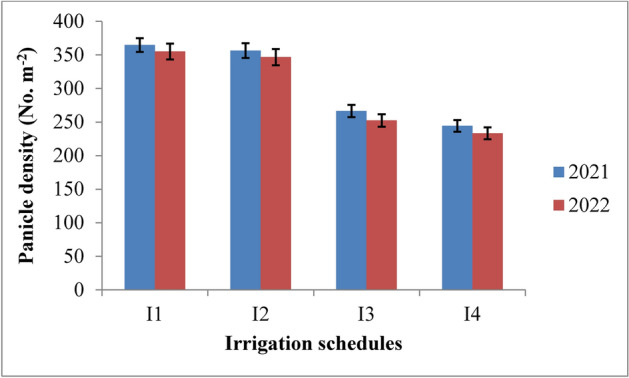
Panicle density of rice as influenced by variable irrigation schedules. (I1: recommended irrigation scheduling; I2: application of irrigation water at field capacity; I3: application of irrigation water at 10% depletion from field capacity; I4: application of irrigation water at 20% depletion from field capacity). The vertical bars represent the mean ± SEm values.

**Fig. 9:**
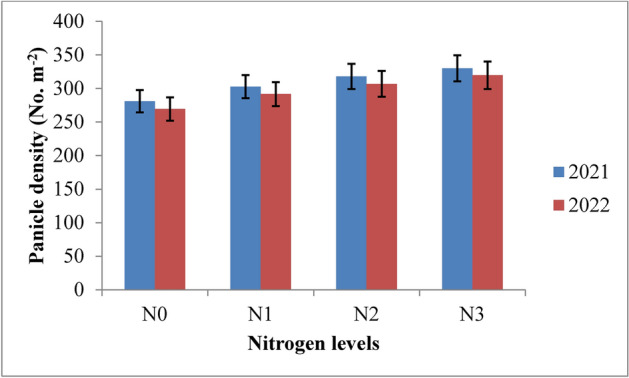
Panicle density of rice as influenced by varying nitrogen levels. {(N0: Control; N1: 75% RDN (Recommended dose of nitrogen); N2: 100% RDN; N3: 125% RDN}. The vertical bars represent the mean ± SEm values.

The irrigation regimes and nitrogen levels had a significant impact on rice yield attributes in both 2021 and 2022 (Table 6). Panicle density was notably influenced by irrigation schedules and nitrogen levels during both years of the study (Figs. 8 and 9). The RIS (I1) achieved the highest panicle density, though it was statistically similar to the field capacity treatment (I2). Both outperformed the 10% depletion (I3) and 20% depletion (I4) treatments. The recommended irrigation schedule recorded the highest panicle density of 364.5 and 355.0 in 2021 and 2022, respectively, followed closely by the field capacity treatment, with densities of 356.3 and 346.6 at harvest. In contrast, the 20% depletion treatment (I4) yielded the lowest panicle densities of 244.1 and 233.1 during the two years. The superiority of the RIS and field capacity treatment over the 10% depletion treatment was 26.96% and 25.28% in 2021, and 28.92% and 27.20% in 2022. Nitrogen levels also significantly affected panicle density across both years. The application of 125% of the recommended nitrogen dose (RDN) resulted in the highest panicle density, though it was statistically similar to the 100% RDN treatment at harvest. The 125% RDN treatment recorded panicle densities of 330.0 and 319.6 at the panicle initiation stage, followed by the 100% RDN treatment with densities of 317.7 and 306.7 in 2021 and 2022, respectively. The superiority of 125% RDN and 100% RDN over the 75% RDN treatment was 8.30% and 4.75% in 2021, and 8.79% and 4.95% in 2022. The control treatment (N0) consistently resulted in the lowest panicle densities during both years. All interaction effects between treatments were found to be non-significant.

**Table 6:** Influence of variable irrigation schedules and nitrogen levels on yield attributes of rice.

Treatments	Panicle density (No. m^−2^)		Panicle length (cm)		Panicle weight (g)		Spikelets/panicle		Filled grains per panicle		1000-grain weight (g)	
	2021	2022	2021	2022	2021	2022	2021	2022	2021	2022	2021	2022
Irrigation schedules												
I_1_	364.5	355.0	23.89	23.20	2.89	2.80	107.64	103.89	101.96	99.17	25.15	24.34
I_2_	356.3	346.6	23.18	22.37	2.78	2.69	103.42	98.61	97.78	94.95	24.91	24.03
I_3_	266.2	252.3	20.25	19.28	2.56	2.46	87.28	83.36	78.51	75.54	24.03	23.06
I_4_	244.1	233.1	19.85	18.78	2.33	2.21	81.61	77.42	72.07	68.94	22.02	20.99
SE(m) ±	5.38	7.33	0.21	0.27	0.09	0.10	1.27	2.20	1.27	1.54	0.10	0.07
C.D. (*p* ≤ 0.05)	18.98	25.88	0.74	0.85	0.32	0.37	4.50	6.77	4.51	5.44	0.36	0.32
Nitrogen levels												
N_0_	280.9	269.3	20.49	19.61	2.41	2.31	83.31	79.39	74.80	71.87	23.47	22.54
N_1_	302.6	291.5	21.50	20.62	2.57	2.47	90.37	86.45	82.61	79.69	23.90	22.97
N_2_	317.7	306.7	22.23	21.95	2.72	2.62	97.75	93.83	90.67	87.74	24.24	23.32
N_3_	330.0	319.6	22.94	22.05	2.87	2.77	103.51	99.60	98.23	95.30	24.51	23.58
SE(m) ±	4.96	5.15	0.26	0.05	0.08	0.10	1.94	1.98	2.48	2.50	0.09	0.05
C.D. (*p* ≤ 0.05)	14.58	15.12	0.76	0.14	0.24	0.32	5.88	5.79	7.58	7.64	0.28	0.26

{I1: recommended irrigation scheduling; I2: application of irrigation water at field capacity; I3: application of irrigation water at 10% depletion from field capacity; I4: application of irrigation water at 20% depletion from field capacity; N0: Control; N1: 75% RDN (Recommended dose of nitrogen); N2: 100% RDN; N3: 125% RDN}.

#### Panicle length

Panicle length in rice was significantly influenced by both irrigation schedules and nitrogen levels throughout the 2021 and 2022 experiments. Among the irrigation treatments, the RIS (I1) resulted in the longest panicle length, though it was statistically on par with the field capacity treatment (I2). Both of these irrigation regimes were superior to the 10% (I3) and 20% (I4) depletion from field capacity treatments. The highest panicle lengths of 23.89 cm and 23.20 cm were recorded under I1 in 2021 and 2022, respectively, while the I2 treatment followed closely with lengths of 23.18 cm and 22.37 cm. The lowest panicle lengths, 19.85 cm in 2021 and 18.78 cm in 2022, were observed with the 20% depletion treatment (I4). Compared to the 10% depletion treatment, the RIS and field capacity treatments showed improvements in panicle length of 15.23% and 12.64% in 2021, and 16.89% and 13.81% in 2022, respectively. Nitrogen application also had a significant effect on panicle length. The 125% recommended nitrogen dose (RDN) achieved the greatest panicle length, though it was statistically similar to the 100% RDN treatment at harvest in both years. In 2021, the 125% RDN treatment recorded a panicle length of 22.94 cm, while the 100% RDN treatment measured 22.23 cm. Similarly, in 2022, the panicle lengths were 22.05 cm for the 125% RDN and 21.95 cm for the 100% RDN treatment. The 125% and 100% RDN treatments demonstrated an advantage over the 75% RDN treatment, with increases of 6.27% and 3.28% in 2021, and 6.48% and 6.05% in 2022, respectively. The control treatment (N0) consistently recorded the shortest panicle length in both years. No significant interaction effects were found between the different irrigation and nitrogen treatments.

#### Panicle weight

The study revealed that rice panicle weight was notably influenced by both irrigation schedules and nitrogen levels across the 2021 and 2022 growing seasons. Among the various irrigation approaches, the recommended irrigation schedule (I1) produced the highest panicle weights, which were comparable to those achieved with irrigation at field capacity (I2). In contrast, irrigation treatments with 10% and 20% depletion from field capacity (I3 and I4) resulted in lower panicle weights. Specifically, the RIS yielded panicle weights of 2.89 g and 2.80 g, while field capacity provided weights of 2.78 g and 2.69 g in 2021 and 2022, respectively. The lowest panicle weights, 2.33 g and 2.21 g, were observed with 20% depletion from field capacity. Compared to the 10% depletion treatment, the recommended schedule and field capacity treatments were superior by 11.41% and 7.91% in 2021, and 12.14% and 8.55% in 2022. Different levels of nitrogen also significantly impacted panicle weight. The highest weights were achieved with 125% of the recommended nitrogen dose (RDN), though this level was similar to the 100% RDN treatment. Specifically, 125% RDN resulted in panicle weights of 2.87 g and 2.77 g, whereas 100% RDN yielded 2.72 g and 2.62 g in 2021 and 2022, respectively. Both 125% RDN and 100% RDN were significantly better than 75% RDN, showing improvements of 10.45% and 5.51% for 125% RDN, and 10.83% and 5.72% for 100% RDN in the two years. The control group (N0) had the lowest panicle weights in both years. Interaction effects between irrigation and nitrogen treatments were not significant.

#### Spikelets per panicle

The study demonstrated that both irrigation schedules and nitrogen levels significantly influenced the number of spikelets per panicle in rice over the 2021 and 2022 growing seasons. Among the various irrigation strategies, the RIS (I1) achieved the highest number of spikelets per panicle, comparable to field capacity irrigation (I2). In contrast, irrigation at 10% and 20% depletion from field capacity (I3 and I4) resulted in fewer spikelets. Specifically, the recommended irrigation yielded 107.64 and 103.89 spikelets per panicle, while field capacity produced 103.42 and 98.61 spikelets in 2021 and 2022, respectively. The lowest counts of 81.61 and 77.42 spikelets were observed with 20% depletion. The recommended schedule and field capacity treatments surpassed the 10% depletion treatment by 18.91% and 15.60% in 2021, and 19.76% and 15.46% in 2022. Regarding nitrogen levels, the application of 125% of the recommended dose (RDN) resulted in the highest number of spikelets per panicle, though it was similar to the 100% RDN treatment. Specifically, 125% RDN yielded 103.51 and 99.60 spikelets, while 100% RDN produced 97.75 and 93.83 spikelets in 2021 and 2022, respectively. Both 125% RDN and 100% RDN were significantly more effective than 75% RDN, showing improvements of 12.69% and 7.54% for 125% RDN, and 13.20% and 7.86% for 100% RDN. The control treatment (N0) had the lowest number of spikelets per panicle in both years.

#### Filled grains per panicle

**Fig. 10:**
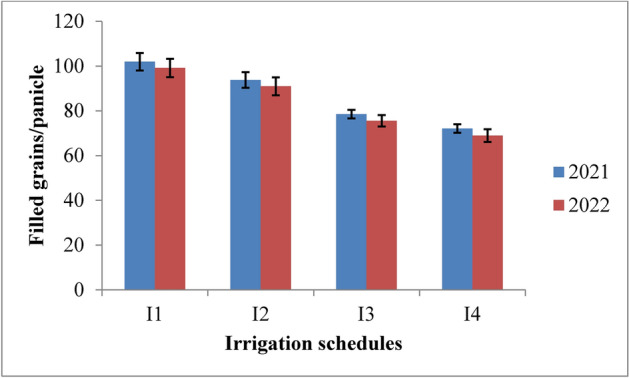
Filled grains/panicle of rice as influenced by variable irrigation schedules (I1: recommended irrigation scheduling; I2: application of irrigation water at field capacity; I3: application of irrigation water at 10% depletion from field capacity; I4: application of irrigation water at 20% depletion from field capacity). The vertical bars represent the mean ± SEm values.

**Fig. 11:**
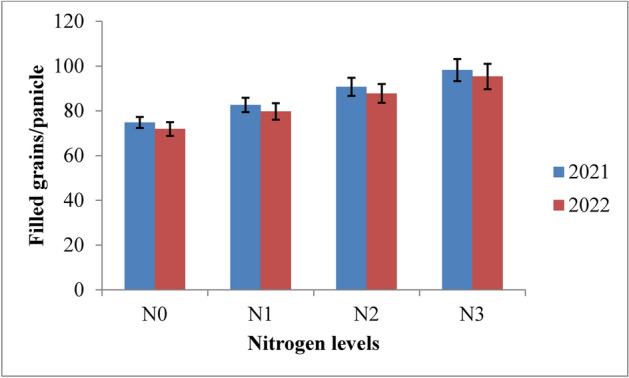
Filled grains/panicle of rice as influenced by varying nitrogen levels. {(N0: Control; N1: 75% RDN (Recommended dose of nitrogen); N2: 100% RDN; N3: 125% RDN}. The vertical bars represent the mean ± SEm values.

The analysis of data from the experiment revealed that both irrigation schedules and nitrogen levels had a significant impact on the number of filled grains per panicle in rice across the 2021 and 2022 growing seasons (Figs. 10 and 11). Among the irrigation schedules tested, the RIS (I1) consistently resulted in the highest number of filled grains per panicle, comparable to the field capacity irrigation (I2). In contrast, treatments with 10% and 20% depletion from field capacity (I3 and I4) yielded fewer filled grains. Specifically, the recommended irrigation schedule achieved 101.96 and 99.17 filled grains per panicle, while the field capacity treatment produced 97.78 and 94.95 filled grains in 2021 and 2022, respectively. The lowest counts of filled grains, 72.07 and 68.94, were observed with the 20% depletion treatment. Compared to the 10% depletion treatment, the recommended schedule and field capacity treatments demonstrated improvements of 22.99% and 19.70% in 2021, and 23.82% and 20.44% in 2022. In terms of nitrogen levels, the application of 125% of the recommended dose (RDN) resulted in the highest number of filled grains per panicle, though this was similar to the 100% RDN treatment. Specifically, 125% RDN recorded 98.23 and 95.30 filled grains, whereas 100% RDN yielded 90.67 and 87.74 filled grains in 2021 and 2022, respectively. Both 125% RDN and 100% RDN were significantly superior to 75% RDN, showing improvements of 15.90% and 8.88% for 125% RDN, and 16.37% and 9.17% for 100% RDN. The control treatment (N0) exhibited the lowest number of filled grains per panicle in both years. Interaction effects between different treatments were not significant.

#### Test weight

The test weight of rice was notably affected by both irrigation schedules and nitrogen levels during the 2021 and 2022 growing seasons. Among the various irrigation methods, RIS (I1) resulted in the highest test weights, similar to those achieved with field capacity irrigation (I2). On the other hand, irrigation treatments with 10% and 20% depletion from field capacity (I3 and I4) resulted in lower test weights. Specifically, the RIS achieved test weights of 25.15 g and 24.34 g, while the field capacity treatment yielded 24.91 g and 24.03 g in 2021 and 2022, respectively. The lowest test weights of 22.02 g and 20.99 g were observed with 20% depletion from field capacity. The recommended irrigation and field capacity treatments showed a significant advantage over the 10% depletion treatment, with improvements of 13.20% and 15.45% in 2021, and 14.91% and 14.99% in 2022. Regarding nitrogen application, the highest test weights were achieved with 125% of the recommended dose (RDN), although these results were comparable to those obtained with 100% RDN. Specifically, 125% RDN produced test weights of 24.51 g and 23.58 g, while 100% RDN resulted in 24.24 g and 23.32 g in 2021 and 2022, respectively. Both 125% RDN and 100% RDN treatments were significantly better than the 75% RDN treatment, with improvements of 10.76% and 5.44% for 125% RDN, and 11.05% and 5.71% for 100% RDN. The control treatment (N0) had the lowest test weight in both years. There were no significant interaction effects between the different treatments.

### Grain, straw, biological yield and harvest index

**Fig. 12:**
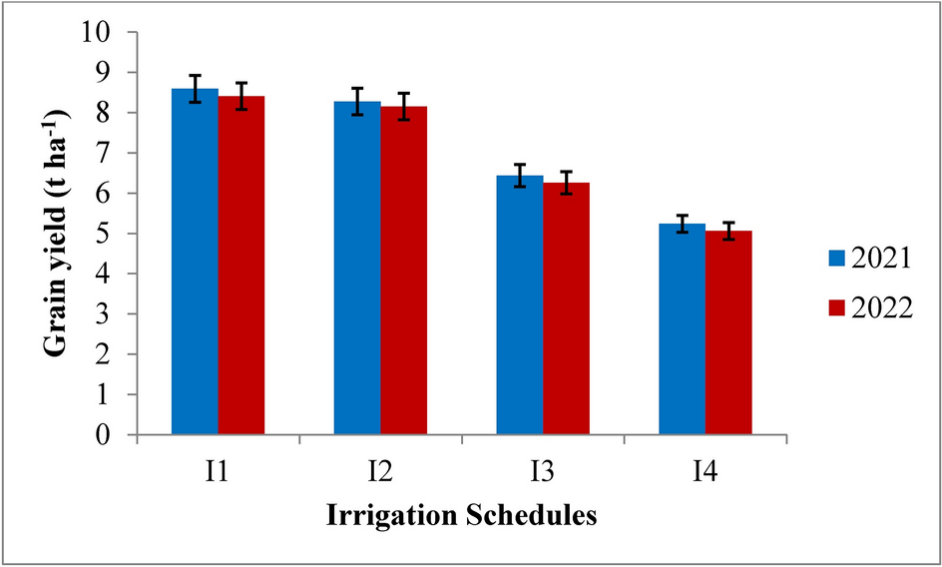
Grain yield of rice as influenced by variable irrigation schedules. (I1: recommended irrigation scheduling; I2: application of irrigation water at field capacity; I3: application of irrigation water at 10% depletion from field capacity; I4: application of irrigation water at 20% depletion from field capacity). The vertical bars represent the mean ± SEm values.

**Fig. 13:**
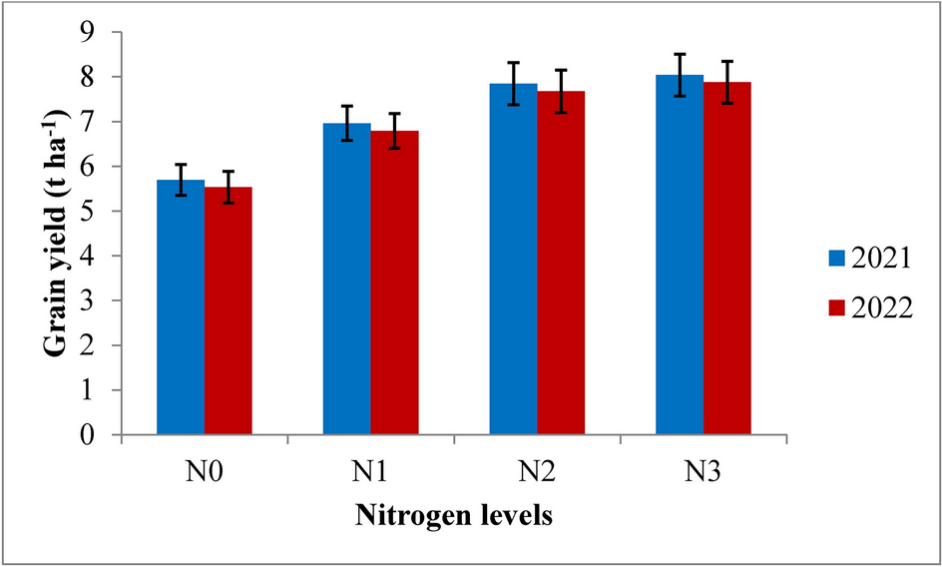
Grain yield of rice as influenced by varying nitrogen levels. {(N0: Control; N1: 75% RDN (Recommended dose of nitrogen); N2: 100% RDN; N3: 125% RDN}. The vertical bars represent the mean ± SEm values.

**Fig. 14:**
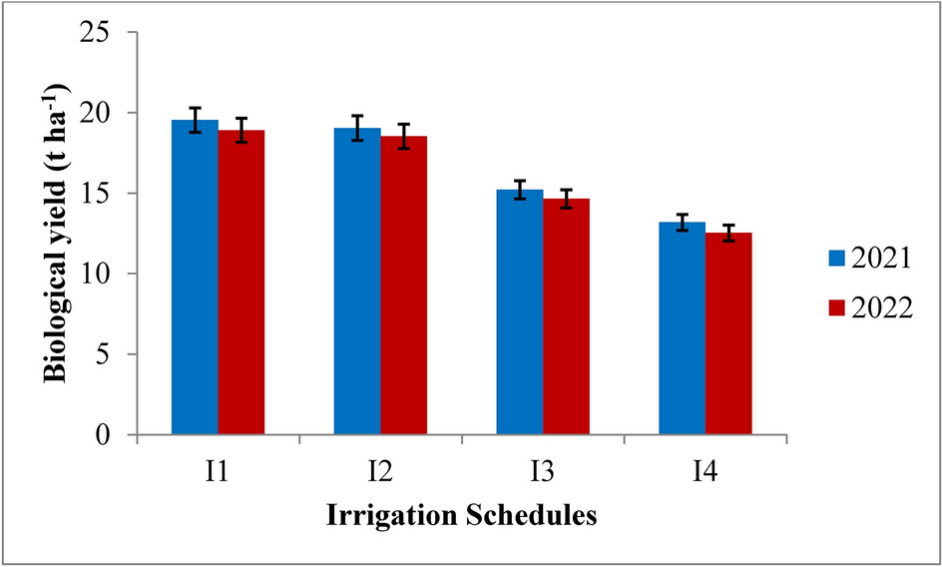
Biological yield of rice as influenced by variable irrigation schedules. (I1: recommended irrigation scheduling; I2: application of irrigation water at field capacity; I3: application of irrigation water at 10% depletion from field capacity; I4: application of irrigation water at 20% depletion from field capacity). The vertical bars represent the mean ± SEm values.

**Fig. 15:**
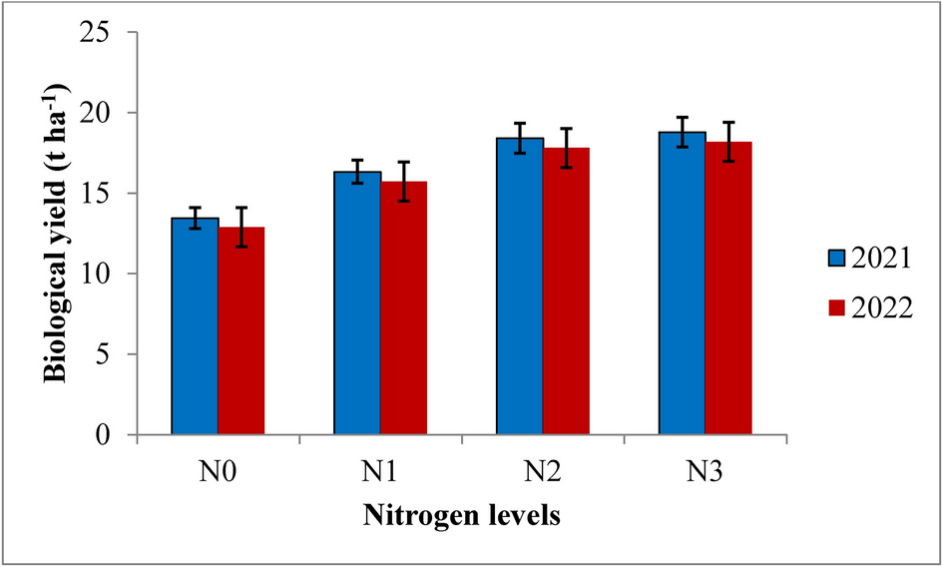
Biological yield of rice as influenced by varying nitrogen levels. {(N0: Control; N1: 75% RDN (Recommended dose of nitrogen); N2: 100% RDN; N3: 125% RDN}. The vertical bars represent the mean ± SEm values.

The grain, straw, biological yield and harvest index were significantly (*p* ≤ 0.05) influenced by the variable irrigation schedules and nitrogen levels (Table 7 and Figs. 12, 13, 14 and 15). Among the variable irrigation schedules, RIS (I_1_) recorded significantly higher grain yield compared to I_3_ and I_4_ treatments during 2021 and 2022 but was at par with the application of irrigation water at field capacity (I_2_). The recommended irrigation schedule achieved the highest grain yields of 8.58 t/ha and 8.40 t/ha, followed closely by the field capacity treatment with yields of 8.27 t/ha and 8.15 t/ha during 2021 and 2022, respectively, whereas the lowest grain yield of 5.23 t ha^−1^ and 5.05 t ha^−1^ at harvest was recorded with the application of irrigation water at 20% depletion from field capacity, respectively, during both the years of experimentation. The superiority exhibited by RIS and field capacity treatment over application of irrigation water at 10% depletion from field capacity was 25.1% and 22.2% during 2021 and 25.6% and 23.3% during 2022 respectively. The data revealed that different nitrogen levels had significant effect on the grain yield of rice during both the years. Among the different nitrogen levels, 125% RDN (recommended dose of nitrogen) recorded the highest grain yield; however, it was at par with 100% RDN treatment during both the years of experimentation. Application of nitrogen @ 125% recommended dose recorded significantly higher grain yield of 8.03 t ha^−1^ and 7.87 t ha^−1^, followed by 100% RDN treatment which recorded the grain yield of 7.84 t ha^−1^ and 7.67 t ha^−1^ during 2021 and 2022 respectively. The superiority exhibited by 125% RDN and 100% RDN over 75% RDN was 13% and 11% during 2021 and 14% and 12% during 2022 respectively. Moreover, it was found that (N_0_) control treatment recorded significantly lowest grain yield during both the years of experimentation.

**Table 7:** Influence of variable irrigation schedules and nitrogen levels on yield and harvest index of rice.

Treatments	Grain yield (t ha^−1^)		Straw yield (t ha^−1^)		Biological yield (t ha^−1^)		Harvest index (%)	
	2021	2022	2021	2022	2021	2022	2021	2022
Irrigation schedules								
I_1_	8.58	8.40	10.94	10.49	19.52	18.90	43.95	44.44
I_2_	8.27	8.15	10.76	10.37	19.03	18.52	43.45	44.00
I_3_	6.43	6.25	8.77	8.38	15.20	14.64	42.30	42.69
I_4_	5.23	5.05	7.94	7.46	13.18	12.52	39.68	40.33
SE(m) ±	0.26	0.24	0.33	0.28	0.36	0.32	0.23	0.25
C.D. (*p* ≤ 0.05)	0.86	0.76	0.88	0.82	1.08	1.02	0.67	0.72
Nitrogen levels								
N_0_	5.69	5.53	7.75	7.35	13.44	12.88	42.33	42.93
N_1_	6.95	6.78	9.36	8.92	16.32	15.71	42.58	43.15
N_2_	7.84	7.67	10.56	10.12	18.40	17.80	42.60	43.08
N_3_	8.03	7.87	10.74	10.31	18.78	18.18	42.75	43.28
SE(m) ±	0.24	0.22	0.28	0.24	0.28	0.26	0.24	0.31
C.D. (*p* ≤ 0.05)	0.82	0.74	0.81	0.78	0.82	0.75	0.74	0.92

{I1: recommended irrigation scheduling; I2: application of irrigation water at field capacity; I3: application of irrigation water at 10% depletion from field capacity; I4: application of irrigation water at 20% depletion from field capacity; N0: Control; N1: 75% RDN (Recommended dose of nitrogen); N2: 100% RDN; N3: 125% RDN}.

Analysis of the data revealed that straw yield of rice was significantly affected by irrigation schedules and nitrogen levels during both the years of experimentation. Among the variable irrigation schedules, RIS (I1) recorded significantly highest straw yield but was at par with the application of irrigation water at field capacity (I2) as compared to 10% depletion from field capacity (I3) and 20% depletion from field capacity (I4) treatments during both the years (2021 and 2022) of experimentation. The highest straw yield of 10.94 t ha^−1^ and 10.49 t ha^−1^ was found in recommended irrigation scheduling followed by field capacity treatment which recorded the grain yield of 10.76 t ha^−1^ and 10.37 t ha^−1^ at harvest during 2021 and 2022 respectively whereas the lowest straw yield of 7.94 t ha^−1^ and 7.46 t ha^−1^ at harvest was recorded with the application of irrigation water at 20% depletion from field capacity respectively during both the years of experimentation. The superiority exhibited by recommended irrigation scheduling and field capacity treatment over application of irrigation water at 10% depletion from field capacity was 19.83% and 18.49% during 2021 and 20.11% and 18.97% during 2022 respectively.Among the different nitrogen levels, 125% RDN (recommended dose of nitrogen) recorded the highest straw yield; however, it was at par with 100% RDN treatment at harvest of rice crop during both the years of experimentation. 125% RDN recorded significantly higher straw yield of 10.74 t ha^−1^ and 10.31 t ha^−1^ at harvest followed by 100% RDN treatment which recorded the straw yield of 10.56 t ha^−1^ and 10.12 t ha^−1^ during 2021 and 2022 respectively. The superiority exhibited by 125% RDN and 100% RDN over 75% RDN was 12.84% and 11.36% during 2021 and 13.48% and 11.85% during 2022 respectively. Moreover, it was found that (N0) control treatment recorded significantly lowest straw yield during both the years of experimentation. All the interaction effects of different treatments were found to be non-significant.

RIS treatment recorded significantly highest biological yield but was at par with the application of irrigation water at field capacity as compared to 10% depletion from field capacity and 20% depletion from field capacity treatments during both the years of experimentation. The highest biological yield of 19.52 t ha^−1^ and 18.90 t ha^−1^ was found in RIS followed by field capacity treatment which recorded the biological yield of 19.03 t ha^−1^ and 18.52 t ha^−1^ during 2021 and 2022, respectively, whereas the lowest biological yield of 13.18 t ha^−1^ and 12.52 t ha^−1^ was recorded with the application of irrigation water at 20% depletion from field capacity, respectively during both the years of experimentation. Among the different nitrogen levels, 125% RDN recorded the highest biological yield, however, it was at par with 100% RDN treatment at harvest during both the years of experimentation. 125% RDN recorded significantly higher biological yield of 18.78 t ha^−1^ and 18.18 t ha^−1^ followed by 100% RDN treatment which recorded the biological yield of 18.40 t ha^−1^ and 17.80 t ha^−1^ during 2021 and 2022, respectively. Moreover, it was found that (N_0_) control treatment recorded significantly lowest biological yield during both the years of experimentation.

Harvest index of rice was significantly affected by irrigation schedules and nitrogen levels during both the years of experimentation. Among the variable irrigation schedules, recommended irrigation scheduling (I1) recorded significantly highest harvest index but was at par with the application of irrigation water at field capacity (I2) as compared to 10% depletion from field capacity (I3) and 20% depletion from field capacity (I4) treatments during both the years (2021 and 2022) of experimentation. The highest harvest index of 43.95 and 44.44 was found in RIS followed by field capacity treatment which recorded the harvest index of 43.45 and 44.00 at harvest during 2021 and 2022 respectively whereas the lowest harvest index of 39.68 and 40.33 at harvest was recorded with the application of irrigation water at 20% depletion from field capacity respectively during both the years of experimentation. Among the different nitrogen levels, 125% RDN (recommended dose of nitrogen) recorded the highest harvest index; however, it was at par with 100% RDN treatment at harvest of rice crop during both the years of experimentation. 125% RDN recorded significantly higher harvest index of 42.75 and 43.28 at harvest followed by 100% RDN treatment which recorded the harvest index of 42.60 and 43.08 during 2021 and 2022 respectively. Moreover, it was found that (N0) control treatment recorded significantly lowest harvest index during both the years of experimentation. All the interaction effects of different treatments were found to be non-significant.

#### Relative economics

The data pertaining to total cost of cultivation, gross returns, total returns, net returns and benefit cost ratio is presented in Table 8 and was pooled for 2021 and 2022 crop growing seasons. The relative economics was worked out taking into consideration, the cost of production for each treatment, the corresponding marketable yield with prevalent prices per unit output. It is evident from the pooled data that highest net returns were realized when irrigation was applied at field capacity coupled with the nitrogen application @ 125% RDN (I2N3) during 2021 and 2022. The highest B: C ratio of 1.64 was also obtained with the same combination (I2N3) and the lowest net returns and B: C ratio (0.30) was realized when the irrigation was applied at 20% depletion from field capacity and zero nitrogen application (control), respectively.

**Table 8:** Economics of different treatments (pooled over 2021 and 2022).

Treatment combinations	Total cost of cultivation (₹)	Gross returns from grain yield (₹)	Gross returns from straw yield (₹)	Total returns (₹)	Net returns (₹)	BC ratio
I_1_N_0_	64,300	89,052.17	28,882.5	117,934.7	53,634.7	0.83
I_1_N_1_	65,170	107,076.7	33,635	140,711.7	75,541.7	1.16
I_1_N_2_	65,560	121,721.2	38,740	160,461.2	94,901.2	1.45
I_1_N_3_	65,950	124,017.8	39,355	163,372.8	97,422.8	1.48
I_2_N_0_	58,680	85,245.33	28,229	113,474.3	54,794.3	0.93
I_2_N_1_	59,550	103,406.3	33,470	136,876.3	77,326.3	1.30
I_2_N_2_	59,940	117,834.2	38,265	156,099.2	96,159.2	1.60
I_2_N_3_	60,330	120,477.5	38,850	159,327.5	98,997.5	1.64
I_3_N_0_	57,480	64,915.5	23,942.5	88,858	31,378.0	0.55
I_3_N_1_	58,350	79,302.17	28,944	108,246.2	49,896.2	0.86
I_3_N_2_	58,740	91,433.33	30,806	122,239.3	63,499.3	1.08
I_3_N_3_	59,130	94,293.33	31,258.5	125,551.8	66,421.8	1.12
I_4_N_0_	57,160	52,652.17	21,601	74,253.17	17,093.2	0.30
I_4_N_1_	58,030	67,600	25,689	93,289	35,259.0	0.61
I_4_N_2_	58,420	72,407.83	28,322	100,729.8	42,309.8	0.72
I_4_N_3_	58,810	74,925.5	28,862	103,787.5	44,977.5	0.76

{I1: recommended irrigation scheduling; I2: application of irrigation water at field capacity; I3: application of irrigation water at 10% depletion from field capacity; I4: application of irrigation water at 20% depletion from field capacity; N0: Control; N1: 75% RDN (Recommended dose of nitrogen); N2: 100% RDN; N3: 125% RDN}.

## Discussion

### Plant height

Significantly greater plant height was observed with the recommended irrigation schedule, which was comparable to the field capacity irrigation treatment. Both these treatments demonstrated superior results compared to other irrigation schedules across both years of the experiment. This improvement in plant height can be attributed to the extended availability and absorption of water and nutrients under the recommended and field capacity irrigation conditions, which supported better plant growth. Conversely, deficit irrigation treatments (I3 and I4) led to reduced plant height due to water stress. These results align with those reported by Marimuthu et al.^40^. Among the nitrogen levels tested, the application of 125% of the recommended dose (RDN) resulted in the tallest plants at maturity, although this was comparable to the 100% RDN treatment. Both these nitrogen levels were significantly better than the lower nitrogen treatments and the control. Higher nitrogen levels promoted plant height by enhancing nitrogen availability, which supports cell division and elongation, photosynthesis, and overall growth. This effect is consistent with the findings of Jahan et al.^41^ and Zhang et al.^31^.

### Dry matter accumulation

**Fig. 16:**
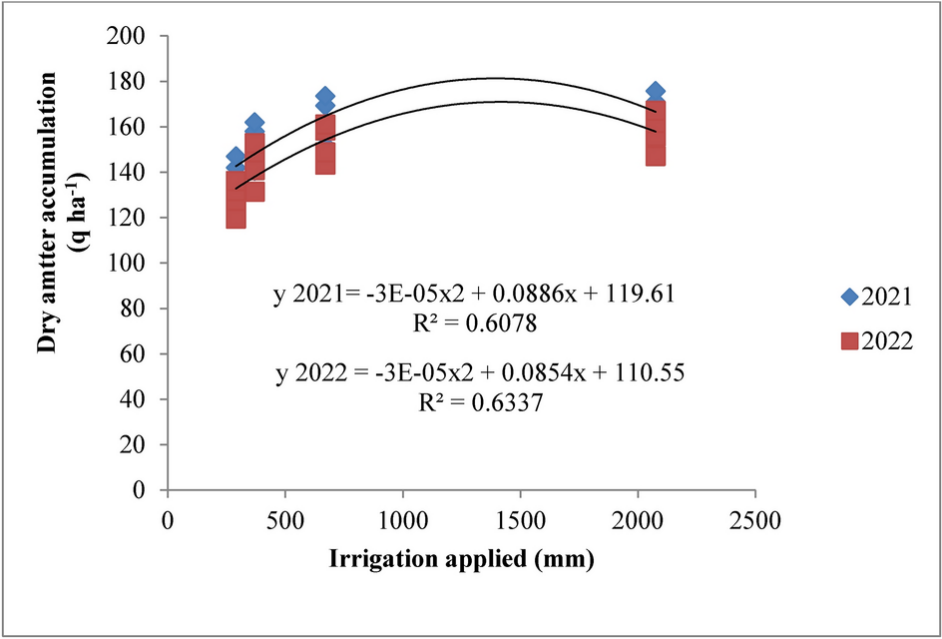
Response of rice dry matter accumulation to irrigation water applied during the two crop growing seasons of 2021 and 2022.

Increased production of total dry matter per unit area is the primary requirement for increased yield. The amount of dry matter produced is dependent on the crop’s capacity for photosynthesis as well as the health of the plant as a whole. The amount of dry matter produced after deducting the amount of photosynthates used for respiration, as well as how the dry matter is distributed among the various plant parts, all affect the economic yield. The results revealed that dry matter accumulation of rice was significantly influenced by variable irrigation schedules and nitrogen levels (Fig. 16). Significantly higher dry matter accumulation was recorded in recommended irrigation scheduling but was at par with application of irrigation water at field capacity. This may be attributed to sufficient supply of water since water was continuously kept above the soil surface throughout the plant cycle resulting in higher water and nutrient uptake as also reported by Kumar et al.^42^. Among different nitrogen levels, 125% RDN (recommended dose of nitrogen) enhanced the dry matter accumulation up to maturity but was at par with 100% RDN and significantly superior over other treatments and control during both the years. Higher dose of nitrogen might have helped in inducing vegetative growth leading to better interception of photosynthetically active radiation, greater photosynthesis by the crop and hence higher dry matter production. These results are in conformity with the findings of Hirzel et al.^43^ and Singh et al.^44^.

### Leaf area index

The leaf area index (LAI) was significantly higher with the recommended irrigation schedule, which was comparable to the field capacity irrigation treatment. Both of these irrigation methods were notably better than other irrigation schedules across both years of the experiment. This improvement is likely due to the sufficient supply of irrigation water, which enhanced nutrient availability and absorption, ultimately increasing leaf area and resulting in a higher LAI. Similar observations were made by Kumar et al.^42^. Regarding nitrogen levels, applying 125% of the recommended dose (RDN) significantly increased the LAI up to 60 days after transplanting (DAT), though it was similar to the 100% RDN treatment. Both levels of nitrogen were significantly better than the lower doses and the control. The enhanced LAI with higher nitrogen levels can be attributed to an increase in the number of leaves, improved leaf development, and extended leaf functionality. These findings align with the research of Salem et al.^45^. Additionally, the increased LAI can be partly explained by the stimulation of cytokinin biosynthesis and its transport from the roots to the aerial parts of the plant, which promotes cell division and increases the number of tillers, thus improving the LAI, as reported by Jalali-Moridani and Amiri^46^.

### Tiller count

**Fig. 17:**
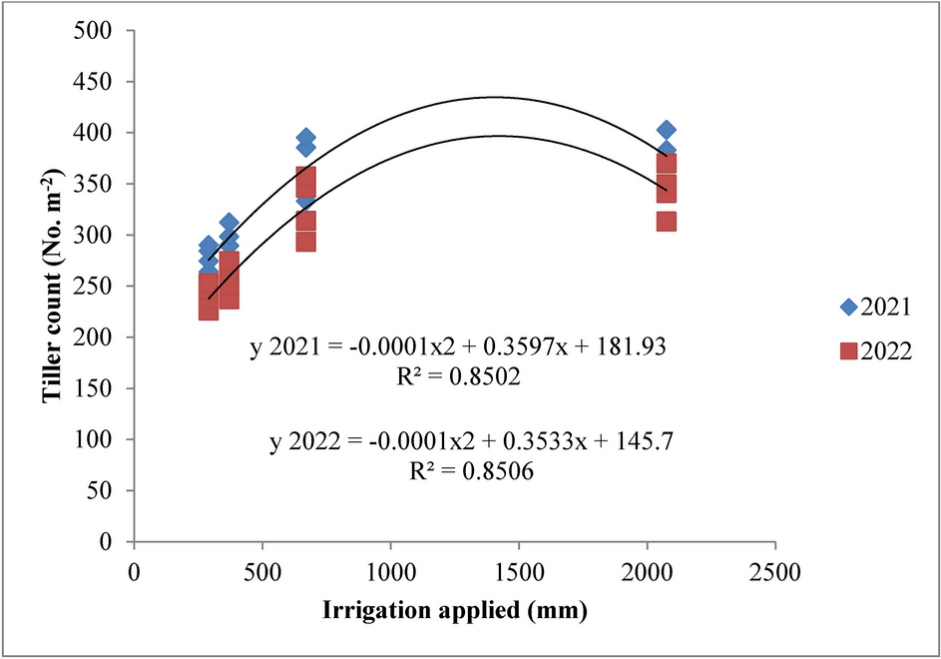
Response of rice tiller count to irrigation water applied during the two crop growing seasons of 2021 and 2022.

The highest tiller count was observed with the recommended irrigation schedule, which was similar to the field capacity irrigation treatment but significantly better than other irrigation schedules across both years of the experiment (Fig. 17). The increased tiller count under these treatments can be attributed to the optimal vegetative growth conditions provided by adequate moisture, which was consistently maintained throughout the crop’s growth. This favourable moisture regime under both recommended and field capacity irrigation enhanced water and nutrient availability, supporting the production of new tillers. In contrast, water stress from deficit irrigation treatments led to reduced tiller numbers due to the death of tillers under such conditions. These results are consistent with the findings of Pandey et al.^47^ and Hasan et al.^48^. Regarding nitrogen levels, applying 125% of the recommended dose (RDN) resulted in the highest tiller count, although it was comparable to the 100% RDN treatment. Both nitrogen levels significantly outperformed lower doses and the control. The increased tiller count with higher nitrogen levels can be attributed to improved nutrient availability, which enhanced the plant’s nutritional status and supported better tillering. These observations align with the research conducted by Singh et al.^44^ and Chen et al.^49^.

### SPAD value

It was observed that SPAD value increased with the advancement of crop age up to 60 DAT and thereafter decreased during both the years of experimentation. Among the variable irrigation schedules, recommended irrigation scheduling recorded significantly highest SPAD value but was at par with the application of irrigation water at field capacity as compared to 10% depletion from field capacity and 20% depletion from field capacity treatments during both the years of experimentation. Sufficient moisture conditions increased SPAD values. This improvement may result from better nutrient and water availability, which enhanced chlorophyll content in leaves^50^. Among the different nitrogen levels, 125% RDN (recommended dose of nitrogen) recorded the highest SPAD value; however, it was at par with 100% RDN treatment throughout the crop growth period during both the years of experimentation. Also, it was found that control treatment recorded significantly lowest SPAD value during both the years of experimentation. Higher SPAD value at elevated nitrogen levels can be credited to more greenness of leaves because of higher chlorophyll content due to better availability of nitrogen, which is indirectly reflected in SPAD values. This is in accordance with the findings of Metwally et al.^51^.

### NDVI value

The recommended irrigation schedule achieved the highest NDVI values, comparable to those from field capacity irrigation, and significantly better than the 10% and 20% depletion treatments in both years of the study. This is likely due to the ample availability of irrigation water, which promoted superior vegetative growth, biomass accumulation, and overall crop health, leading to elevated NDVI values. These findings are consistent with the research by Kimaro et al.^52^. Among the nitrogen levels, the application of 125% of the recommended dose (RDN) resulted in the highest NDVI values, similar to those from the 100% RDN treatment, throughout the growing season in both years. In contrast, the control treatment consistently recorded the lowest NDVI values. The higher nitrogen levels likely enhanced the assimilation of photosynthates into grains and positively influenced other growth characteristics, contributing to higher NDVI readings. These results corroborate those reported by Sen et al.^53^.

### Leaf relative water content

The recommended irrigation schedule yielded significantly higher relative water content (RWC) compared to irrigation treatments with 10% and 20% depletion from field capacity, although it was similar to the field capacity treatment in both years of the study. The superior RWC observed under the recommended and field capacity irrigation treatments can be attributed to the ample water availability, which facilitated greater water uptake by the plants. This observation is consistent with the findings of Zhang et al.^54^. Among the nitrogen levels, the application of 125% of the recommended dose (RDN) resulted in the highest RWC, comparable to the 100% RDN treatment during panicle initiation, 50% flowering, and the milking stages of the rice crop. The control treatment, on the other hand, recorded the lowest RWC in both years. Higher nitrogen levels likely improved RWC by enhancing leaf development and increasing leaf area. Additionally, increased nitrogen promotes root growth, which improves nutrient and water uptake, thereby raising the RWC of rice plants. These results are in line with the research conducted by Umnajkitikorn et al.^55^.

### Yield attributes

**Fig. 18:**
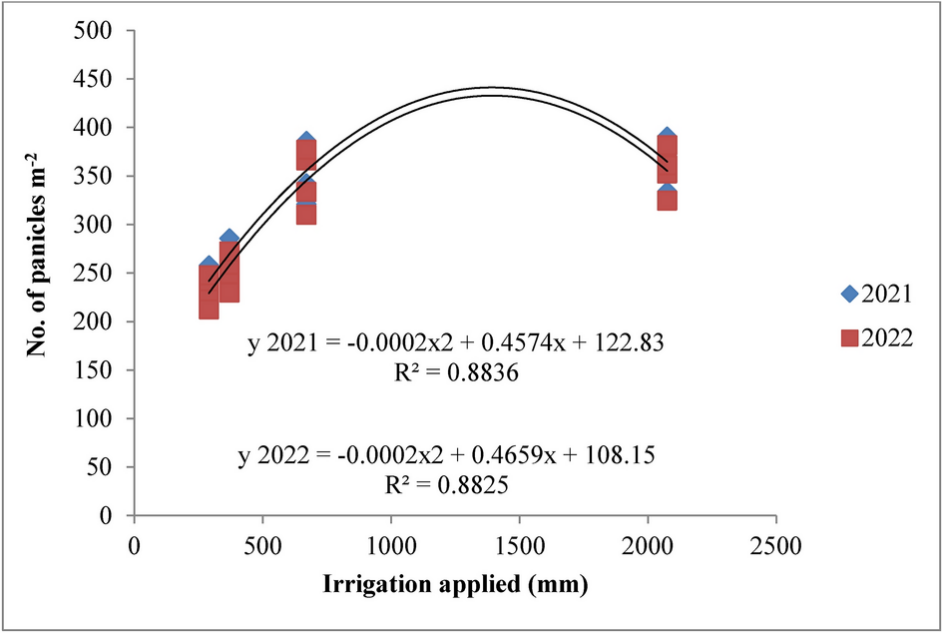
Response of panicle density of rice to irrigation water applied during the two crop growing seasons of 2021 and 2022.

**Fig. 19:**
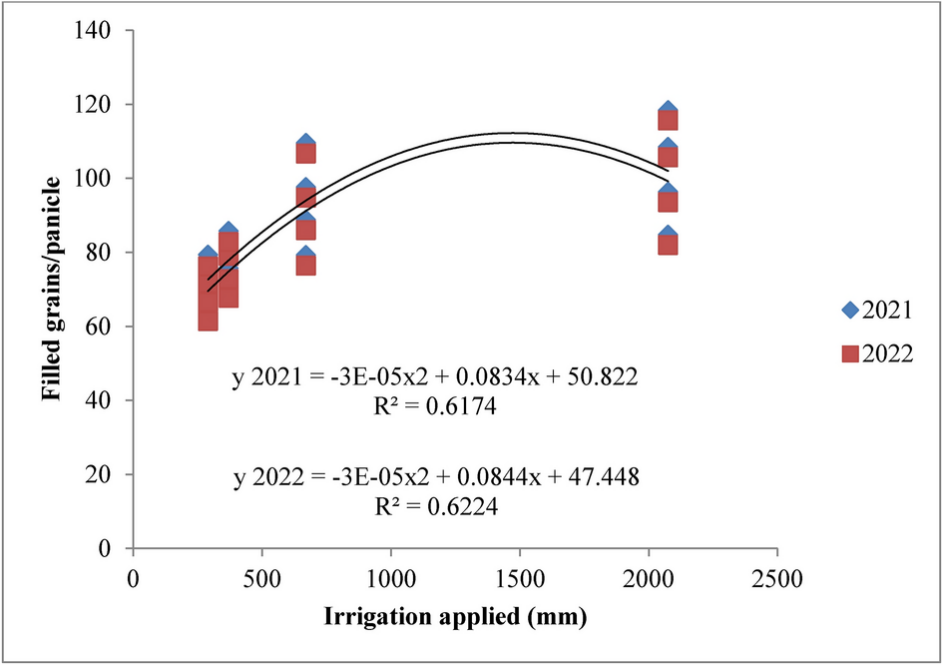
Response of filled grains/panicle of rice to irrigation water applied during the two crop growing seasons of 2021 and 2022.

The important yield attributing characters viz; panicle density, panicle length, panicle weight, spikelets panicle^−1^, spikelets panicle^−1^, number of filled grains panicle^-1^ and test weight showed significant variation under variable irrigation schedules and nitrogen levels (Figs. 18 and 19). The results revealed a significant increase in the panicle density, panicle length, panicle weight, spikelets panicle^−1^, spikelets panicle^−1^, number of filled grains panicle^−1^ and test weight under recommended irrigation scheduling but was at par with application of irrigation water at field capacity and superior over other irrigation regimes. Yield attributing characters were higher under sufficient soil moisture conditions as the crop received enough water without being stressed during the flowering and grain development stages leading to higher water and nutrient uptake. These results are in accordance with the findings of Pandey et al.^56^. The lower values of yield attributes may be due to the fact that plants under moisture stress could not extract more nutrients from the deeper soil layer due to moisture deficit conditions. This ultimately resulted in poor growth, fewer tillers, and subsequently lower values of yield contributing parameters in I3 and I4 treatments. These results are in close conformity with the findings of Sandhu et al.^57^ and Sarkar et al.^58^. Among different nitrogen levels, 125% RDN recorded significantly higher yield contributing characters like panicle density, panicle length, panicle weight, spikelets panicle^−1^, spikelets panicle^−1^, number of filled grains panicle^−1^ and test weight but was at par with 100% RDN and superior over other nitrogen levels and control. Increased values of yield attributing characters was due to the increased availability and uptake of nitrogen, which is a substrate for the synthesis of organic compounds that comprise protoplasm and chlorophyll, cause an increase in cell division and enlargement at higher nitrogen doses. These results are similar to those of Malik et al.^59^ and Bhavana et al.^60^. The lower values of yield attributing characters in control treatment may be the result of insufficient nitrogen for crops to grow and develop more effectively. The findings concur with those of Uddin et al.^61^.

### Grain, straw, biological yield and harvest index

**Fig. 20:**
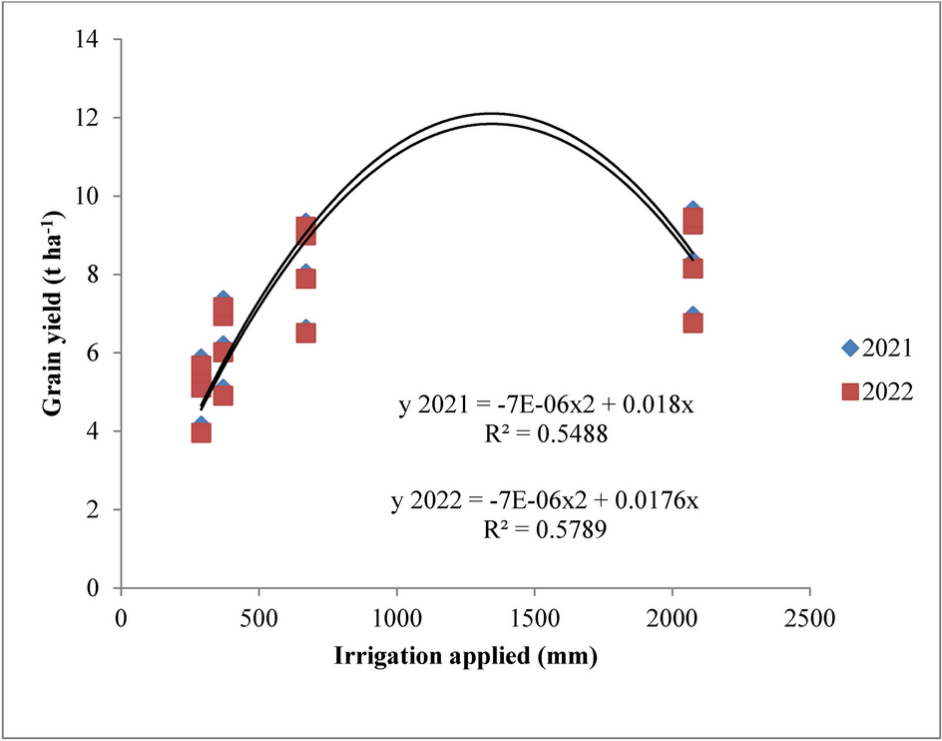
Response of rice grain yield to irrigation water applied during the two crop growing seasons of 2021 and 2022.

**Fig. 21:**
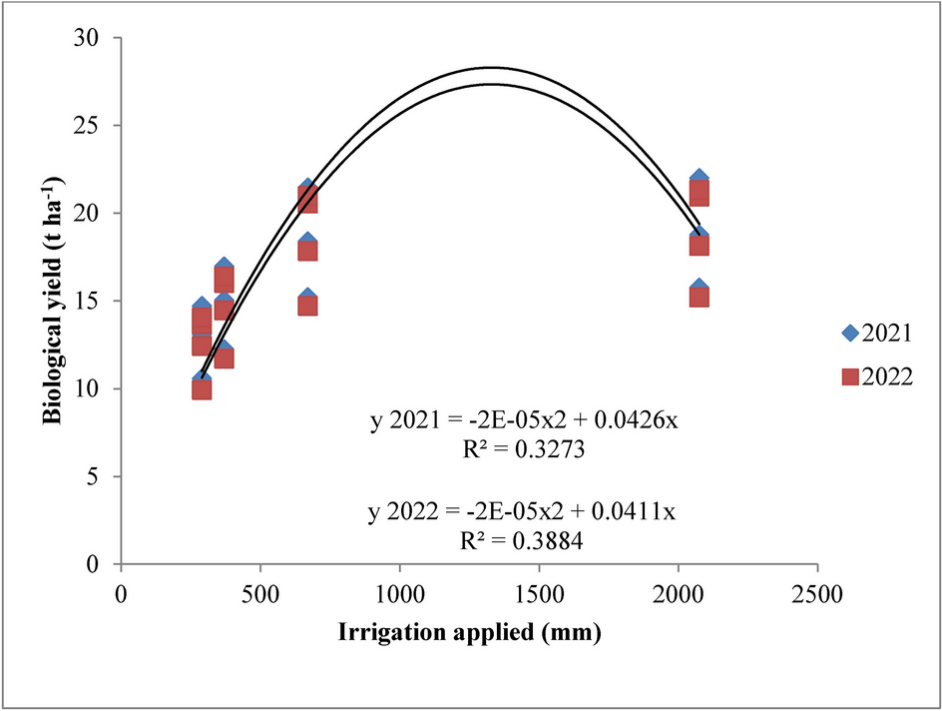
Response of biological yield to irrigation water applied during the two crop growing seasons of 2021 and 2022.

There was a profound influence of the year on the rice yield. The yield was higher in the first year as compared to the second year, due to improved temperature, higher rainfall and sunshine hours. Among the variable irrigation schedules, recommended irrigation scheduling recorded significantly higher grain, straw, biological yield and harvest index but was at par with the application of irrigation water at field capacity and superior over other treatments during 2021 and 2022 (Figs. 20 and 21). The increased yields under recommended irrigation scheduling and application of irrigation water at field capacity might be due to sufficient moisture availability, favourable growing conditions and optimum nutrient supply environment. The favourable growth traits enhanced the yield attributing characters with higher source to sink conversion, which in turn resulted in higher grain yield. These results are in line with the findings of Mir et al.^62^ and Sudhakara et al.^63^ who reported that irrigation maintained at saturation level throughout crop growth period or irrigation at 3 days after disappearance of ponded water registered significantly higher grain and straw yield. The differences in yield among the various irrigation schedules can be attributed to variations in the quantity of applied irrigation water and the corresponding discrepancies in evapotranspiration^64,65^, exerting its influence on the physiological processes^66^. Rice is considered as a major consumer of fresh water resources^67^, requiring more than 3000 L of water to produce 1 kg of grain^68^. Our findings suggest that application of irrigation water at field capacity of soil is better than continuous flooding, resulting in less water requirement and comparable yield. The reason is that in flooded conditions, water is mostly lost as drainage without getting effectively converted into yield. Substantial wastage of water by the farmers through faulty irrigation practices has also been reported by many other researchers^10,69^. This finding aligns with the reports of Dar et al.^70^. Mainuddin et al.^64^ also noted that variations in grain yield are more accurately explained by ETc than by total water applied.

Similarly, the straw and biological yield was higher in the first year compared to the second year. There were also significant differences among the various irrigation regimes. The recommended irrigation scheduling treatment produced the highest biological yield, similar to the application of irrigation water at field capacity, but higher than the yields from treatments with 10% and 20% depletion from field capacity during both years of the experiment. These results clearly indicate that adopting an irrigation strategy that permits moisture depletion below saturation decreases the biological yield, although it also reduces water requirements. The reduction in yield due to soil moisture depletion results from the difficulty in water extraction by roots and the resulting physiological constraints, including faster leaf senescence^71,72^, impairment of the photosynthetic machinery^14^, and a shortened growth cycle. Additionally, it leads to reduced carbon fixation and assimilate translocation as well as lower grain set and development^66^. Application of irrigation water based on depletion from field capacity can be adopted as water saving practices in rice, though with a yield penalty, and keeping the water standing proves to be a yield enhancing practice, but with higher water consumption and difficult to adopt at the initial establishment and the panicle emergence stage, where saturation or submergence is required. Various researchers have explored different yield-enhancing and water-saving irrigation practices to optimize water application^73,74^.

Among the different nitrogen levels, 125% RDN (recommended dose of nitrogen) recorded the highest grain, straw and biological yield and harvest index; however, it was at par with 100% RDN treatment during both the years of experimentation. Control treatment recorded significantly lowest grain, straw and biological yield during both the years of experimentation. The increased yields under higher nitrogen levels may be due to the higher uptake of nitrogen by crop plants which exerted a beneficial effect on chlorophyll content in the leaves, better absorption of nutrients and assimilation from source to sink, which might have enhanced photosynthetic activity, increased dry matter production, enhanced tiller production and yield attributes, thus increased grain and biological yield. These findings are in accordance with the results of Abhinaya et al.^75^, Aparna et al.^76^ and Papia et al.^77^. Yield reduction under lower nitrogen levels and unfertilized control treatment may be due to the reduced uptake of nutrients by the crop plants leading to reduced dry matter production, low tiller production and lesser values of yield contributing characters which ultimately reduced grain, straw, biological yield and harvest index of rice. The results are in conformity with the findings of Zhu et al.^78^ and Xu et al.^79^.

### Relative economics

The efficiency of a treatment or a combination of treatments is finally decided in terms of the economics (benefit: cost ratio) of the treatments. The result of pooled data revealed that highest net returns were realized when irrigation was applied at field capacity coupled with the nitrogen application @ 125% RDN during 2021 and 2022. The highest B: C ratio was also obtained with the same combination and the lowest net returns and B: C ratio was realized when the irrigation was applied at 20% depletion from field capacity and zero nitrogen application (control), respectively. The higher benefit cost ratio in field capacity water regime was attributed to higher grain and straw yield leading to higher gross and net returns with reduced cost of cultivation compared to flooding (I1). These results are in accordance with findings of Brar et al.^80^ and Hou et al.^81^. Increasing levels of nitrogen progressively enhanced the grain yield and straw yield resulting in higher gross returns, net returns and benefit: cost ratio. Similar results of higher economic returns with elevated N levels were reported by Murthy et al.^82^ and Mishra et al.^83^.

## Conclusion

To achieve sustainable rice production, it is crucial to reduce water losses and enhance water productivity. While new irrigation techniques and tools are effective in monitoring water use and improving yields and water productivity for various crops, their application in rice, especially in economically disadvantaged regions, remains limited. Our study used profile probe moisture sensors to track moisture dynamics and assess water balance in rice cultivation. We found that continuous flooding and excessive water depth are not necessary for rice growth. Irrigating at field capacity was the most effective treatment for optimal growth and yield. In water-scarce areas, field capacity irrigation can achieve minimal yield loss while maintaining productivity. In situations where water is limited, irrigation at field capacity can be used with minimal yield losses. Our results indicated that the highest growth and productivity was observed in flooded irrigation treatment and application of irrigation water at field capacity. Based on these findings, it is recommended to irrigate the soil at field capacity in order to get optimum growth and productivity and to adopt sensor-based irrigation strategies for effective moisture monitoring and water conservation, with minimal or no impact on yield. We also observed that the application of nitrogen @ 100% RDN improved the growth, productivity and profitability of rice under temperate conditions of Kashmir valley.

## Supplementary Information

Supplementary Information.

## Data Availability

The datasets used and/or analyzed during the current study are available from the corresponding author on reasonable request.
