# Supplementary material for: Optimizing irrigation and nitrogen levels to achieve sustainable rice productivity and profitability
Source: Sci Rep. 2025 Feb 24;15:6675. doi: 10.1038/s41598-025-90464-8 (PMC11850893; doi:10.1038/s41598-025-90464-8)
Supplement: Supplementary file 1 — Supplementary Information. [file 41598_2025_90464_MOESM1_ESM.docx]

**Supplementary Table 1: Irrigation details:**

| **Treatments** | **Irrigation amount (2021)** | **Irrigation amount (2022)** |
| --- | --- | --- |
| I_1_:recommended irrigation scheduling | 2150 mm | 2000 mm |
| I_2_:at field capacity | 660 mm | 680 mm |
| I_3_:10 % depletion from field capacity | 360 mm | 380 mm |
| I_4_:20 % depletion from field capacity | 280 mm | 300 mm |
